# Enhanced heat tolerance of viral-infected aphids leads to niche expansion and reduced interspecific competition

**DOI:** 10.1038/s41467-020-14953-2

**Published:** 2020-03-04

**Authors:** Mitzy F. Porras, Carlos A. Navas, James H. Marden, Mark C. Mescher, Consuelo M. De Moraes, Sylvain Pincebourde, Andrés Sandoval-Mojica, Juan A. Raygoza-Garay, German A. Holguin, Edwin G. Rajotte, Tomás A. Carlo

**Affiliations:** 1 Department of Entomology, Pennsylvania State University, University Park, PA 16802 USA; 2 Department of Physiology, Instituto de Biociências, Universidade de São Paulo, Butanta, 05508090 São Paulo Brazil; 3 Department of Biology, The Pennsylvania State University, University Park, PA 16802 USA; 4 Department of Environmental Systems Science, ETH Zürich, 8092 Zürich, Switzerland; 5 Institut de Recherche sur la Biologie de l’Insecte, UMR 7261, Université de Tours, 37200 Tours, France; 6 Citrus Research and Education Center, University of Florida, Lake Alfred, FL 33850 USA; 7 Department of Medicine, University of Toronto, Toronto, ON Canada; 8 Departamento de Ingeniería Eléctrica, Universidad Tecnológica de Pereira, Pereira, Colombia

**Keywords:** Ecology, Behavioural ecology, Ecophysiology, Population dynamics, Zoology

## Abstract

Vector-borne pathogens are known to alter the phenotypes of their primary hosts and vectors, with implications for disease transmission as well as ecology. Here we show that a plant virus, barley yellow dwarf virus, increases the surface temperature of infected host plants (by an average of 2 °C), while also significantly enhancing the thermal tolerance of its aphid vector *Rhopalosiphum padi *(by 8 °C). This enhanced thermal tolerance, which was associated with differential upregulation of three heat-shock protein genes, allowed aphids to occupy higher and warmer regions of infected host plants when displaced from cooler regions by competition with a larger aphid species, *R. maidis*. Infection thereby led to an expansion of the fundamental niche of the vector. These findings show that virus effects on the thermal biology of hosts and vectors can influence their interactions with one another and with other, non-vector organisms.

## Abstract (web summary)

Organisms living on and inside of plants—such as microbes and herbivorous insects—can interact in complex ways. Here the authors show that a plant virus increases the temperature of the plant and also the thermal tolerance of an aphid species feeding on the plant; this change in thermal tolerance also affects competition with another aphid species.

## Introduction

Vector-borne pathogens can enhance their transmission by altering the phenotypes of their primary hosts and vectors in ways that influence the outcome of interactions between them^1–4^. Pathogens may directly manipulate vector behavior, for example, but they can also alter the quality of resources that primary hosts provide to vectors, as well as host-derived sensory cues that mediate vector recruitment and dispersal^5–8^. Research exploring such effects has primarily focused on implications for disease transmission^2–4^; however, pathogen effects on the phenotypes of hosts and vectors can also influence the ways in which these species interact with other organisms^1^. Yet, despite increasing recognition that pathogens and other microbial symbionts often have profound effects on the phenotypes of their hosts, the implications of such effects for interspecific and community-level interactions are only beginning to be explored^9–13^.

An extensive literature addresses the evolution of host manipulation by parasites^2–8,10–15^, and increased attention has recently focused on the manipulative effects of vector-transmitted microbial pathogens^14,15^. It is now clear that pathogens frequently influence host–vector interactions both through direct effects on vectors (e.g., via alteration of feeding behaviors^16–18^) and through effects on primary hosts that influence host–vector interactions^2–5^. For example, there is evidence from both animal and plant pathogen systems that infection can alter host odors in ways that enhance vector attraction^2–4,19–22^. Furthermore, pathogen effects on host physiological traits, such as plant nutritional and defense chemistry, can alter the quality of infected hosts as a resource for the vector and thereby influence the initiation and duration of feeding, rates of development and reproduction, and the timing of dispersal^2,21–23^.

While it can be difficult to ascertain the adaptive significance of specific pathogen effects on host and vector phenotypes (i.e., to conclusively distinguish cases of manipulation from host or vector countermeasures or mere byproducts of pathology)^24^, it is reasonable to assume that effects with implications for pathogen transmission will frequently be under selection^25^. Consequently, it is not surprising that such effects often appear conducive to pathogen transmission^2,3,14–22^. Moreover, the fitness interests of pathogens may align with those of vectors, as it appears likely for viruses that enhance plant quality for aphid vectors and thereby enhance virus acquisition and dissemination^23^. More generally, the relationships between microbial symbionts and their hosts and vectors occur along a continuum ranging from mutualism to parasitism (pathogenicity), and can evolve in response to ecological and natural history factors that influence rates and patterns of transmission^26^. For example, because pathogens depend on the host for survival prior to transmission, the extent to which they exploit their hosts (i.e., their virulence) may be constrained by limited transmission opportunities^27^ or by harsh environmental conditions that compromise host tolerance^26,28^. The latter is consistent with theoretical^27,29^ and empirical evidence^2–5,8,17–22^ showing that interspecific interactions tend to shift from antagonism (e.g., parasitism or competition) toward mutualism or facilitation under more stressful environmental conditions^30^. For instance, some plant viruses that do not rely on vector transmission have been found to enhance the tolerance of host plants to environmental stressors, including drought^28^ and high soil temperatures^26^.

In addition to influencing interactions between hosts and vectors, the effects of a pathogen on vector traits can potentially influence the ways in which they interact with other species. While relatively little work has explored the ecological implications of such effects in vector-borne disease systems^7,14^, there is growing appreciation for the ecological significance of microbial symbionts^12,13^ as well as that of parasitic organisms, including pathogens^31^. Pathogens may affect the structure of ecological communities by suppressing populations of superior competitors (i.e., predators or parasitoids)^32^ or triggering trophic cascades that reduce populations of structurally important species within food webs^33^.

Plant pathogens, in particular, might be expected to have important effects on community dynamics and processes, as plants reside at the center of most terrestrial food webs^27^. Yet, few studies have explored the broader ecological implications of pathogen-induced changes in host and vector phenotypes. Recently, we reported that trophic facilitation between the aphid species *Rhopalosiphum padi* and *R. maidis*—which frequently co-occur on grasses^34–36^—was enhanced by the infection of a shared host plant (spring wheat) by barley yellow dwarf virus (BYDV)^23^. The current study explores how viral infections influence the ecological interactions between *R. padi* and *R. maidis* via virus-mediated effects on the thermal biology of host plants and aphids.

*Rhopalosiphum padi* and *R. maidi*s aphids feed on many of the same plant species and have similar foraging ecology^37^. However, *R. maidis* is approximately twice as large as *R. padi*, which may provide a competitive advantage as large aphids frequently displace smaller ones from common feeding grounds^38^. Furthermore, these two species exhibit different but overlapping thermal tolerance ranges that could influence the partitioning of microhabitats within host plants. The optimal temperature range for development and reproduction of *R. maidis* ranges between 15 and 25 °C^39^, whereas that of *R. padi* falls between 21 and 28 °C^40^. Differences in thermal optima can influence the intensity of competition between aphid species on thermally heterogeneous plant surfaces^41–45^, as aphids regulate their body temperature and reduce exposure to environmental stressors by changing locations^46–48^. Competitive interactions between aphid species resulting from overlap in thermal niche might be further influenced by the effects of viral infection, which has been reported to modify the thermal tolerance of some insects^48,49^. For example, the heat tolerance of the whitefly *Bemisia tabaci* appears to decrease with the tomato yellow leaf curl virus^48^, while the Southern rice black-streaked dwarf virus increases heat tolerance in its vector, *Sogatella furcifera*^49^.

To explore the effects of viral infection on the thermal biology of *R. padi* and *R. maidis*, and resulting effects on their interactions with one another, we conducted laboratory and field experiments using two different strains of BYDV, each of which is transmitted by only one of these aphid species: BYDV-PAV for *R. padi* and BYDV-RMV for *R. maidis*. We investigated how each of these BYDV strains affected (i) the surface temperature profiles of wheat plants, (ii) the spatial distribution of aphids on the host plant, and (iii) aphid heat tolerance (i.e., temperature-mediated effects on key traits, including lifespan, fecundity, and locomotor performance). We found that BYDV-PAV elevates plant surface temperatures and enhances the thermal tolerance of its vector, *R. padi*. Furthermore, this enhanced thermal tolerance had implications for competitive interactions between *R. padi* and *R. maidis*, a larger aphid that typically displaces *R. padi* from host-plant stems to apical leaves, where temperatures are higher. A transcriptomic analysis of virus-free and viruliferous *R. padi* under heat stress indicated that viral infection is associated with the upregulation of three heat-shock protein genes. Taken together, our results show that BYDV alters both the thermal environment and the fundamental niche of its vector, *R. padi*., thereby enhancing the performance of the vector in interspecific competition with *R. maidis*.

## Results

### Infection by BYDV-PAV increases plant temperature

**Fig. 1:**
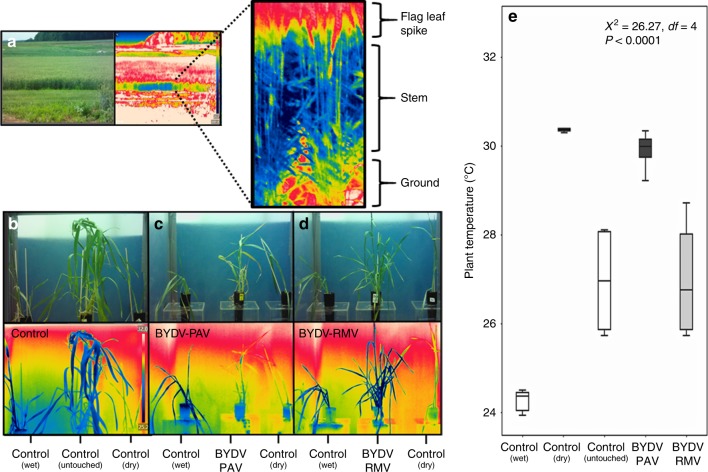
Plant temperature varies with plant region and virus infection status. **a** Thermal images of wheat plants in field, Rock Springs Experimental Station in central PA; upper regions (flag leaves) were significantly warmer than stems (Student’s *t* test = 47.41, *df* = 17, *P* ≤ 0.000; *n* = 20 plants). Thermal images in a rainbow scale, min. temperature in blue = 21.7 °C, max. temperature in white: 25.7 °C. **b**–**e** Virus effects on plant temperature in controlled laboratory experiments. **b**–**d** Thermal imaging of wheat plants in the laboratory experiments. Dry and wet control plants were used as a reference for 0% and 100% transpiration, respectively; untouched control received no manipulation. **e** Temperature of plants infected with BYDV strains, nonparametric ANOVA, Kruskal–Wallis (boxplots display median line, interquartile range (IQR) boxes, 1.5 × IQR whiskers; *n* = 6 plants per treatment). Thermal images in a rainbow scale, min. temperature in blue = 25.2 °C, max. temperature in white: 32 °C. Source data are provided as a Source data file. [Image by Porras and Rajotte].

We used infrared (IR) thermal imaging to characterize the thermal profiles of wheat plants in the field. This revealed that plant stems were consistently cooler (21.08 ± 1.5 °C) than the apical portion of the flag leaf (25.65 ± 1.0 °C) (Fig. 1a). Subsequent laboratory assays conducted in a climate-controlled chamber revealed that infection by BYDV-PAV increased the temperature of both stem and leaf surfaces by 2 to 3 °C, while no similar effects were observed for infection by BYDV-RMV (Fig. 1b–e; Supplementary Fig. 1).

### Interspecific competition and BYDV-PAV infection influence the spatial distribution of *R. padi*

**Fig. 2:**
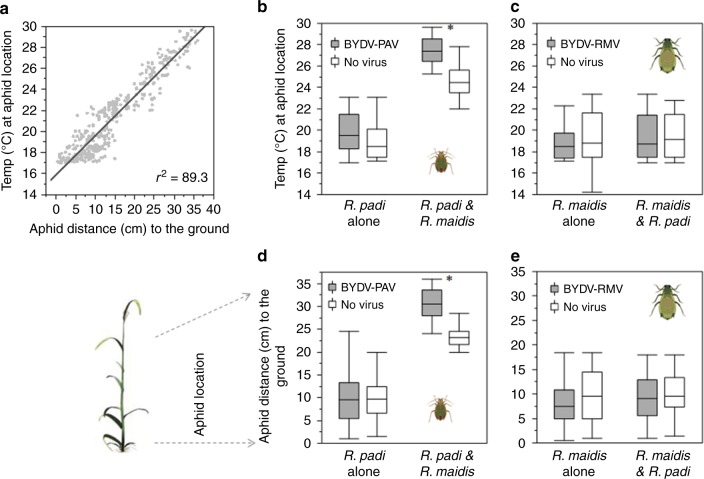
Aphid spatial distribution on infected and uninfected plants in the field for both aphid species. **a** Correlation between plant temperature and plant height. **b**, **c** Temperature at the location occupied by each aphid species, when occurring alone or together, on plants infected by BYDV-PAV (**b**) and BYDV-RMV (**c**). **d**, **e** Distance to ground for locations occupied by each aphid species, when occurring alone or together, on plants infected by BYDV-PAV (**d**) and BYDV-RMV (**e**). Nonparametric ANOVA, Kruskal–Wallis (boxplots display median line, interquartile range (IQR) boxes, 1.5 × IQR whiskers; *n* = 10 plants per treatment), *p < 0.05. Source data are provided as a Source data file.

In a field experiment, we manipulated the presence of both aphids and their respective BYDV strains using a full-factorial design. We observed a tight correlation (*r*^2^ = 89.3) between temperatures at the location of individual aphids and vertical distance to the soil surface (Fig. 2a; Supplementary Table 4). When the two aphid species occurred separately, each exhibited a preference for regions of the stem with temperatures around 18.5 °C (Fig. 2). When the two species co-occurred, however, the distribution of *R. padi* (the smaller aphid) shifted from the stem to the upper leaves, where temperatures were 4 °C higher on average (Fig. 2b–d; Supplementary Tables 4 and 5). In contrast, the distribution of *R. maidis* was unaffected by the presence of *R. padi* (Fig. 2c, e).

Similar displacement of *R. padi* in the presence of *R. maidis* was observed when the two species co-occurred on plants infected with BYDV-PAV (the strain transmitted by *R. padi*), but here *R. padi* moved an average of 2 cm higher than on uninfected plants to a region where temperatures were an average of 6 °C warmer than the stem (Fig. 2b, d). As on uninfected plants, the position of *R. maidis* was not affected by *R. padi* in these trials (Fig. 2c, e). In contrast to BYDV-PAV, infection by BYDV-RMV (the strain transmitted by *R. maidis*) had no apparent effects on aphid spatial distribution (Fig. 2c, e).

### BYDV-PAV infection enhances the thermal tolerance of *R. padi*

**Fig. 3:**
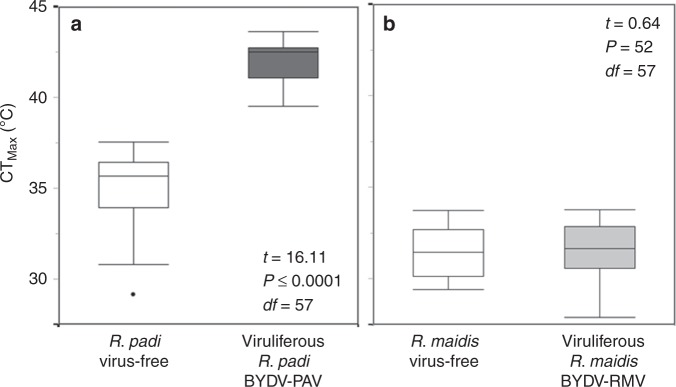
Upper temperature limit for locomotion (CTMax) for viruliferous and virus-free aphid species. CT_Max_ of  (**a**) *R. padi* and (**b**) *R. maidis*. Student’s *t* test (boxplots display median line, interquartile range (IQR) boxes, 1.5 × IQR whiskers; *n* = 30 aphids per treatment). Source data are provided as a Source data file.

We assessed the upper temperature limit for locomotion (CT_Max_) of virus-free and viruliferous individuals of each aphid species by manipulating temperatures in a metal capsule attached to an automated ceramic hotplate (see Methods). The average CT_Max_ of *R. padi* individuals infected with BYDV-PAV was 8 °C higher than that of virus-free individuals (Fig. 3a). In contrast, BYDV-RMV did not significantly affect the CT_Max_ of *R. maidis* (Fig. 3b). Similarly, infection with BYDV-PAV increased the temperature required to cause 50% mortality (LT_50_) in *R. padi*, while BYDV-RMV had no similar effect on the LT_50_ of *R. maidis* (Supplementary Fig. 2). We also examined virus effects on aphid locomotor capacity (assessed as movement speed) across a range of temperatures (14, 22, 26, 18, 30, 34, and 36 °C) in a walk-in climate chamber. At relatively low temperatures (14 and 18 °C), *R. maidis* individuals moved faster than *R. padi* individuals regardless of the infection status (Supplementary Fig. 3). At higher temperatures (26–35 °C), however, viruliferous *R. padi* attained the fastest locomotor speeds (Supplementary Fig. 3). In contrast, viruliferous *R. maidis* exhibited no similar increase in locomotor capacity at high temperatures. Finally, we investigated the thermal preferences of the two aphid species by artificially generating thermal gradients on wheat plants (using electrical resistances, see Supplementary Methods 3). We found that *R. padi* individuals preferred locations that were 3 °C warmer on average than those selected by *R. maidis* individuals; however, these preferences were not affected by viral infection (Supplementary Fig. 4).

### BYDV-PAV infection increases the expression of thermal tolerance genes

**Fig. 4:**
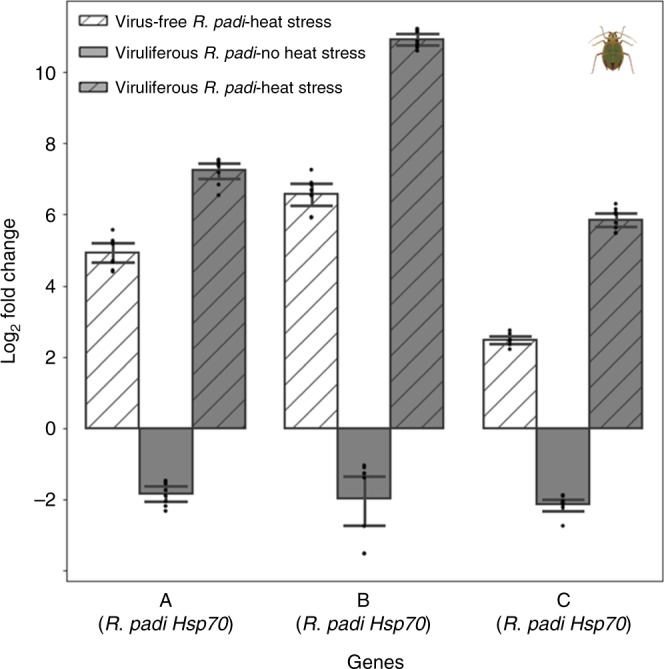
Expression levels of three heat-shock protein genes in *R. padi* individuals differing in infection status and with and without heat stress (at CT_Max_). Expression levels are relative to those observed for virus-free aphids in the absence of heat stress (Hsp70 A: Student’s *t-*test = 9.81, *df* = 34, *P* ≤ 0.0001; Hsp70 B: *t* test = 6.45, *df* = 34, *P* ≤ 0.0001). Values higher than 0 indicate increased gene expression, whereas values lower than 1 indicate gene suppression. Significant fold changes compared with *R. padi* virus-free are indicated, and bars represent mean ± SE (*n* = 8 per treatment). Source data are provided as a Source data file.

To explore potential molecular mechanisms underlying the increased heat tolerance of *R. padi* infected with BYDV-PAV, we extracted messenger RNA (mRNA) from virus-free and viruliferous *R. padi* maintained at room temperature (23 °C) and under heat stress (at CT_Max_). We then constructed transcriptomes for each treatment group using IIlumina Seq. and compared them to determine which genes were up- or down-regulated (see Supplementary Table 2). These analyses identified more than 40 genes that were differentially regulated in aphids exposed to heat stress and BYDV (Supplementary Table 2), 5 of which are known to be associated with stress tolerance. Three of these five genes encode heat-shock proteins that play important roles in heat stress protection in cells (Hsp70 A, B, and C, see Fig. 4), while the other two encode arrestin and a homolog of cubulin^50,51^.

We next used quantitative real-time PCR (qRT-PCR) to measure and compare the expression levels of the three heat-shock proteins and across treatments. Under heat stress, viral infection significantly increased the expression of each of these genes in *R. padi* (Fig. 4), with mRNA levels for Hsp70 increasing by more than 1000-fold, while those of Hsp70 B and Hsp90 C increased by around 100- and 30-fold, respectively. At room temperature (23 °C), Hsp70 A expression in viruliferous aphids was repressed relative to that in uninfected aphids, while levels of the other two genes were unaffected (Fig. 4).

### Temperature and viral infection influence aphid lifespan and fecundity

**Fig. 5:**
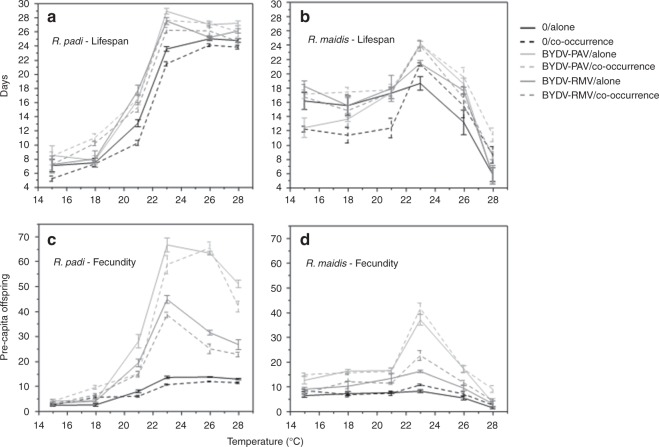
Temperature and viral infection influence lifespan and fecundity of aphid species. Performance curves of lifespan (**a**, **b**) and fecundity (**c**, **d**) of *R. padi* and *R. maidis*, alone or co-occurring with other aphid species, on plants infected with BYDV-PAV or BYDV-RMV and on virus-free plants across a range of environmental temperatures. *Rhopalosiphum padi* transmits BYDV-PAV, while *R. maidis* transmits BYDV-RMV (mean ± SE per treatment per temperature; *n* = 15 per treatment). Source data are provided as a Source data file.

To examine how interspecific competition, temperature, and viral infection influenced the lifespan and fecundity of *R. padi* and *R. maidis*, we conducted a full-factorial experiment with aphids on wheat plants in climate-controlled chambers. In the absence of viral infection or competition between aphid species, increasing air temperatures from 15 to 28 °C tripled the lifespan of *R. padi* while reducing that of *R. maidis* by half (Table 1; Fig. 5a, b; Supplementary Fig. 5; Supplementary Tables 7 and 8). Similarly, fecundity rates for *R. padi* at 28 °C were twice those observed at 15 °C (Fig. 5c), while the fecundity of *R. maidis* dropped by more than half (Fig. 5d). Interspecific competition negatively affected the lifespan and fecundity of both aphid species, although these effects were small relative to the effects of temperature (Table 1). Furthermore, some effects were temperature and species specific (Table 1; Fig. 5). For example, the presence of *R. padi* lowered the lifespan of *R. maidis* by an average of 10 days but only at temperatures between 15 and 21 °C (Fig. 5a). Meanwhile, the presence of *R. maidis* reduced the lifespan of *R. padi* by 2–5 days regardless of temperature (Fig. 5a).

**Table 1:** Analysis of deviance (type II) of full-factorial models from experiments testing the effects of viral infection (virus-free, BYDV-PAV, BYDV-RMV), temperature (15–28 °C), and interspecific co-occurrence (with, without), on the lifespan (days) and fecundity (# of offspring) of *Rhopalosiphum padi* and *R. maidis* raised on wheat plants.

Model effect	*df*	*χ*^2^	*P* value
(A) Response: Lifespan of *R. padi*			
Temp	1	510.23	<0.0001
Virus	2	65.69	<0.0001
Co-occ.	1	9.76	0.0018
Temp:virus	2	3.31	0.1909
Temp:co-occ.	1	3.94	0.0471
Virus:co-occ.	2	6.70	0.0349
Temp:virus:co-occ.	2	5.39	0.0673
(B) Response: Fecundity of *R. padi*			
Temp	1	378.81	<0.0001
Virus	2	236.05	<0.0001
Co-occ.	1	2.15	0.1421
Temp:virus	2	7.25	0.0265
Temp:co-occ.	1	0.61	0.4317
Virus:co-occ.	2	0.78	0.6762
Temp:virus:co-occ.	2	0.59	0.7438
(C) Response: Lifespan of *R. maidis*			
Temp	1	1.44	0.2295
Virus	2	11.08	0.0039
Co-occ.	1	0.45	0.4998
Temp:virus	2	3.37	0.1850
Temp:co-occ.	1	1.89	0.1684
Virus:co-occ.	2	2.82	0.2439
Temp:virus:co-occ.	2	8.34	0.0155
(D) Response: Fecundity of *R. maidis*			
Temp	1	11.49	0.0007
Virus	2	172.04	<0.0001
Co-occ.	1	0.11	0.7342
Temp:virus	2	3.35	0.1872
Temp:co-occ.	1	0.09	0.7592
Virus:co-occ.	2	1.46	0.4804
Temp:virus:co-occ.	2	0.76	0.6815

Remarkably, viral infection had positive effects on the lifespan and fecundity of both *R. padi* and *R. maidis*, although these effects were again temperature and species specific (Fig. 5a–d). At 23 °C, the lifespan of *R. padi* increased by 20% on BYDV-PAV-infected plants and 12.5% on BYDV-RMV-infected plants compared to their uninfected controls, while that of *R. maidis* increased by 50% and 31% on these treatments, respectively (Fig. 5a, b). The magnitude of virus effects on fecundity was much greater than those on lifespan. For instance, at 23 °C, average fecundity increased by 320% for *R. padi*, and by 230% for *R. maidis* (Fig. 5c, d) on plants infected with either strain of BYDV. These positive effects of viral infection on lifespan and fecundity tended to mitigate the negative effects of interspecific competition (Fig. 5; Supplementary Fig. 6).

## Discussion

The results of our field and laboratory experiments demonstrate that BYDV-PAV both increases the surface temperature of host plants and confers heat tolerance to its vector *R. padi*. Meanwhile, no similar effects were apparent for BYDV-RMV and its vector, *R. maidis*. Increased heat tolerance in viruliferous *R. padi* was associated with the upregulation of heat-shock protein genes and allowed *R. padi* to make use of formerly unsuitable regions of host plants, thereby expanding its fundamental thermal niche. Furthermore, viral infection led to large increases in the fecundity and lifespan of aphids under heat stress conditions—especially for *R. padi*—while also ameliorating the negative effects of interspecific competition.

In the absence of interspecific competition, both aphid species preferred to occupy cooler stem microhabitats on the lower mid-section of plants, regardless of viral infection (Fig. 2). When the two species co-occurred, however, *R. padi* moved from the stems to the upper leaves of the plant where temperatures were higher (Figs. 1 and 2). While an increase in overall aphid density might influence the outcome of competition, our experimental design sought to minimize effects of resource limitation and initial crowding (see Methods). Together with the clear spatial segregation of the two species, this suggests that the observed shift in the location of *R. padi* when co-occurring with *R. maidis* is most likely due to interference competition, consistent with previous observations showing that larger aphids frequently displace smaller competitors from common feeding grounds^38^. When *R. padi* and *R. maidis* co-occurred on plants infected by BYDV-PAV, *R. padi* moved to even higher—and thus warmer—positions than those occupied on virus-free plants (Figs. 1 and 2).

The mechanisms underlying the observed increase in surface temperatures of wheat plants infected with BYDV-PAV (Fig. 1) are unknown, but it is possible that BYDV-PAV disrupts leaf stomata, as has been shown for tobacco plants infected with tobacco mosaic virus^52^, perhaps due to membrane depolarization caused by salicylic acid induction in response to viral infection, leading to turgor loss in the guard cells^51^. The absence of such effects in BYDV-RMV (the strain vectored by the *R. maidis*) might then potentially be explained by the comparatively lower replication rate of this strain in host plants^53^.

In addition to raising the surface temperature of infected plants, BYDV-PAV conferred a marked increase in thermal tolerance to *R. padi* (Fig. 3), likely explaining the ability of this aphid to occupy higher positions on plants infected by BYDV-PAV when competing with *R. maidis* (Fig. 2). Our analyses of *R. padi* transcriptomes revealed patterns consistent with this enhanced heat tolerance. Specifically, we found a higher expression of the Hsp genes in viruliferous vs. virus-free *R. padi* under heat stress (Fig. 4; Supplementary Table 2). These genes are responsible for the repair and degradation of proteins damaged by high temperatures, thus promoting the maintenance of cell homeostasis^54,55^. A previous study^56^ showed that BYDV binds to an Hsp (GroEL) of symbiotic mutualistic bacteria (*Buchnera* sp.) that live in the *R. padi* gut, raising the possibility that binding of BYDV-PAV to Hsps of the symbiont, or of the aphid itself, might influence Hsp expression. However, further research is needed to elucidate mechanisms by which BYDV-PAV alters aphid gene expression and to establish a link between such effects and aphid thermal tolerance.

The elevated temperature that BYDV-PAV causes in host plants and the corresponding enhancement of thermal tolerance in its vector, *R. padi*, may have implications both for virus transmission and for the ecological interactions of the host plant and vector with other organisms. Previous work has shown that BYDV infection can enhance plant nutritional quality for aphids^23^, which may explain the positive effects of both strains of BYDV on aphid lifespan and fecundity we documented in our experiments (Fig. 5; Supplementary Fig. 6). Such effects, in turn, may favor virus transmission by enhancing the rate at which viruliferous aphids disperse from infected plants (e.g., due to crowding caused by rapid population growth). The effects of BYDV-PAV on the thermal biology of both host plant and vector may have similarly important consequences for virus transmission. In addition to enhancing the performance of *R. padi* in competition with other aphids such as *R. maidis*, such effects might also alter interactions between *R. padi* and other organisms such as aphid mutualists (e.g., aphid-tending ants) and antagonists (e.g., predators and parasitoids), with potentially important implications for food webs and communities that remain to be explored. Such interactions may be of particular interest in light of ongoing changes in global climate, which raise urgent questions about the ways in which organisms cope with thermal stressors and implications of warming for ecological communities.

To conclude, our findings document the strong effects of a plant virus both on the surface temperature profile of host plants and on the thermal physiology and life histories of aphid vectors. These effects result in the expansion of the fundamental niche of one of the vectors with the consequence of reducing the effects of interference competition between congeneric species. Our work thus adds to the growing body of literature revealing the remarkable capacity of viral pathogens and other microbial symbionts to modify the phenotypes of their multicellular hosts, and the broader implications of such effects on structure and dynamics of populations and communities.

## Methods

### Aphid colonies and experimental plants

Stock aphid colonies were initiated using individuals kindly provided by Dr. S. Gray (Laboratory of Virology, Cornell University), and maintained at low density on barley plants (*Hordeum vulgare*) in a greenhouse at 20 ± 2 °C and ambient photoperiod. All aphids used in experiments were kept in small colonies (~12 aphids per plant) to avoid stress on individuals associated with high population densities. Wheat plants (*Triticum aestivum* L.) were grown from seed in trays (SS-SC Greenhouse Megastore, CA, USA) of greenhouse cones (4 cm diameter × 21 cm long) (SC10 Greenhouse Megastore; ~100 seeds per tray) with a standard substrate including macro- and micro-nutrients (Premier Pro-Mix, Quakertown, PA, USA). Plants were watered twice a day and kept in a growth chamber at 22 ± 1 °C with a 16 h photoperiod (430 µmol m^−2^ s^−1^), 8 h dark period at 20 ± 1 °C, and a relative humidity (RH) of 60% at the Entomology Department of Penn State University.

### Producing viruliferous aphids and virus-infected plants

Virus strains BYDV-PAV and BYDV-RMV were also provided by Dr. S. Gray. The transmission of each virus strain is species specific, BYDV-PAV for *R. padi* and BYDV-RMV for *R. maidis*. This strain specificity is mediated by the interaction between viral capsid proteins with aphid-specific receptors^57^. Aphids were experimentally infected by allowing them to feed for 3 days on black oat leaves (*Avena strigosa*) infected with the appropriate virus strain. Plants were infected by moving viruliferous aphids to healthy wheat plants and allowing them to feed for 12 days. The aphids were then removed, and viral infection was confirmed on each plant using a double-antibody sandwich, enzyme-linked immunosorbent assay (DAS-ELISA) (Agdia Inc., Elkhart, IN, USA). Absorbance was measured at 30-min intervals using a micro-titer plate reader-spectrophotometer (Spectramax 190, Molecular Devices, Silicon Valley, CA) with Agdia-positive and -negative controls on each plate. Samples with absorbance values (*A*_405_) three times higher than those of healthy plants were considered positive for viral infection. Virus titer for infected plants and aphids were estimated from the ELISA absorbance values following the protocol in Jiménez-Martínez et al.^58^, which allows the conversion of the absorbance into a virus titer concentration using a standard curve of known concentrations of purified virus (50, 100, 500, and 1000 ng), healthy plant sap, and BYDV-infected plant.

### Virus effects on plant temperature

Thermal characterization of virus-free wheat plants under field conditions: we characterized the natural temperature profiles of wheat plants (5 weeks old) in a cultivated field at the Penn State Russell E. Larson Agricultural Research Center in Central Pennsylvania (USA). For 20 randomly selected plants, we measured the average temperatures of stems and apical flag leaves, using an IR thermal camera (T650SC; FLIR Inc., Wilsonville, OR, USA) with a 25 mm lens (15° field of view) positioned perpendicularly 1 m from the plant (three pictures per plant) from 15:00 to 15:30 h, solar elevation 60.5° in June 2015. We obtained measurements of the temperature of the stem and flag leaf from the IR image as follows: in the IR image, we traced a vertical line in the middle of the plant from the base of the stem to the apical part of the flag leaf, using the ResearchIR software (FLIR systems, USA). We then extracted 12 temperature data points, six on the stem and six on the flag leaf, from the middle of each plant. Temperature points were located ten pixels apart (1.0 cm). Temperature differences between stems and apical leaves were compared using Student’s *t* tests.

Thermal characterization of virus-free and virus-infected plants under controlled conditions: 2e tested the effects of viral infection on plant temperatures in a controlled climate chamber (Conviron CMP 3244, 1.15 m × 2.5 m × 2 m) at a light intensity of 94.17 W m^−2^ and 50% RH. These experiments employed a full-factorial design to assess effects of viral infection (at three levels: virus-free plants [untouched control that received no manipulation], BYDV-PAV-infected plants, and BYDV-RMV-infected plants) and temperature (at three levels: 15, 23, and 28 °C); each factor combination was replicated on six different plants. Wheat plants (4 to 5 weeks old) were acclimated to each treatment for 24 h. Thermal images were obtained between 9:00 a.m. and 11:00 a.m., using the IR Thermal Camera as above. Imaging protocols were modified from Leinonen and Jones^59^. Because a plausible mechanism by which virus infection might alter plant temperature is by disrupting leaf stomata^51^ and thereby altering transpiration rates, we included the wet and dry controls as reference points approximating the temperatures plant leaves would attain under conditions of zero and maximum transpiration, respectively, in the same environment. Each image included a wet control (plant sprayed with water) and dry control (leaves and stems coated with Vaseline) to provide reference points approximating the temperatures that plants would attain under conditions of zero and maximum transpiration, respectively, in the same environment.

Temperature measurements were obtained similarly to previous experiments, but in this case, for 15 points (five each at the base of the stem, second tiller, and flag leaf). We used the average temperature of these points in our analysis. Temperature differences among the treatments were analyzed using nonparametric analysis of variance (ANOVA), Kruskal–Wallis, followed by nonparametric multiple comparisons (Wilcoxon’s method) because the data did not satisfy normality assumptions.

### Interspecific competition and BYDV-PAV infection influence the spatial distribution of *R. padi*

Spatial distribution of aphids: To examine how the distribution of aphids on a plant (microhabitat choices) was affected by interspecific competition and viral infection, we conducted a field experiment using a raised bed with natural soil (120 cm × 100 cm × 40 cm, 50 cm above ground) at the Pennsylvania State University Horticultural Facility (University Park, PA, USA). Wheat seeds were planted at 1 seed per 4.5 cm^2^ density. We used a 2 × 2 full-factorial experiment to test for the effects of BYDV (with and without) and aphid co-occurrence (one or two species) on the distribution of *R. padi* and *R. maidis* on host plants. Each factor combination was replicated ten times using randomly selected plants and aphid individuals. For treatments using a single aphid species, we placed six adult aphids on the stem of a wheat plant. For interspecific co-occurrence treatments, we placed 12 aphids on the stem of each plant, six from each species as in the single-species treatment. Initial aphid densities thus differed between plants with one or two aphid species; however, we used relatively large plants (50 cm, 6 weeks old) to minimize potential effects of resource limitation. Aphids were individually placed on plant stems (6 cm above the soil surface) using a paintbrush and allowed to settle before the next aphid was released (with at least 1 min between releases) to minimize the chance of dispersal due to initial crowding. Plants were enclosed in a transparent acrylic tube (4.5 cm diameter × 35 cm length) that had two windows (3 cm × 5 cm) covered with Lumite fabric (OHCO, Georgia, GA, USA). Air temperature inside these enclosures was not significantly different than outside air temperature at the time the experiments were conducted (+0.2 °C, Student’s *t* test = 0.22, *df* = 77.55, *P* = 0.8251). After 24 h, we examined the distribution of the aphids on the plant and measured (i) the plant surface temperature at each aphid’s location using a thermocouple thermometer with RS232 output data logger (VWR, Radnor, PA, USA) and (ii) the vertical distance from each individual to the ground. We removed the acrylic tube, measured the plant temperature, and collected the aphids (the trial reading took 4 h, starting at 9:00 a.m.). Then, the viral infection of plants and aphids was confirmed using DAS-ELISA. Temperature differences between species across aphid locations were analyzed using two-way ANOVAs.

### Virus effects on thermal tolerance of aphids

Identification of the upper thermal limit (CT_Max_) of virus-free and viruliferous aphids: To determine (CT_Max_) for virus-free and viruliferous aphids of each species, we employed a protocol modified from that of Ribeiro et al.^60^, using a hotplate with a programmable heating rate controlled by a computer interface (Sable Systems, LV, USA). The temperature was monitored by two independent thermocouple channels connected to a TC2000 Thermocouple Meter (Sable Systems)^53^. One thermocouple was attached to the surface of the hotplate, and the other sensor was attached inside the metal container (aluminum clear glass top container 3.2 cm diameter × 2 cm height, Eslinger & Co. Inc., MN, USA) in which we placed an individual aphid. This equipment was located inside an automated thermal chamber (dimensions of incubator’s cabin: width 40.5 cm × 35 cm length × 40 cm height). We transferred an individual aphid (4-day-old; aphids were grown on 20 different plants per infection treatment) onto the metal pelt and exposed it to increasing temperature at a rate of 0.1 °C min^−1^ until its locomotion stopped. CT_Max_ was recorded when the aphid turned upside down and could no longer return to the upright position within 5 s. The aphid was returned to a plant for recovery. Data points were only considered valid if the aphid displayed normal activity 2 h after a CT_Max_ test^53^. The aphid was then immediately frozen in liquid nitrogen and stored at −80 °C. Aphid infection status was then confirmed using DAS-ELISA as described above (Supplementary Table 1). CT_Max_ virus-free and viruliferous aphids were compared using Student’s *t* tests. Additional physiological parameters, including lethal thermal dose 50, locomotor capacity and behavioral thermal preference, were assessed using methods described in the Supplementary Methods 1–3.

### Changes in gene expression associated with thermal tolerance in *R. padi*

We used a paired design to measure the differences in gene expression of virus-free and viruliferous *R. padi* exposed to thermal stress (at CT_Max_). As factors we had two groups: viral infection (virus-free and viruliferous aphids) and temperature (room temperature [23 °C] and heat stress [CT_Max_]), with three replicates per combination of factors. A replicate trial consisted of 20 (4-day-old) adult aphids, which were collected from different host plants (15 plants per virus treatment). Each aphid was flash frozen in liquid nitrogen immediately after reaching its CT_Max_ (as previously determined using an earlier experiment) and stored at −80 °C for RNA extraction. RNA was extracted using the RNeasy^®^ Mini Kit (Qiagen Inc., Valencia, CA, USA), according to protocols specified by the manufacturer, and was quantified using a NanoDrop 1000 spectrophotometer (Thermo Fisher Scientific, Wilmington, DE, USA) and Bioanalyzer Nano assay (Agilent). Sample trials were sequenced across three lanes on the Illumina HiSeq 2500 platform. Libraries were made with the Illumina TruSeq Stranded mRNA Library Prep Kit, according to the manufacturer’s protocols. The paired-end sequencing resulted in 1 × 150 bp reads. Differential expression analysis was performed using Trimmomatic v.0.3^61^, Tophat^62^, and Cufflinks^63^ packages (for details see Supplementary Methods 4 and Supplementary Table 2).

qRT-PCR was used to quantify the expression levels of candidate genes identified from the transcriptomic analysis: We performed another trial of the CT_Max_ experiment as described above; measurements of candidate gene expression levels were normalized to actin and ribosomal protein S8 (*n* = 8) (primer sequences are given in Table S3) following a protocol modified from Fussnecker et al.^64^. For more details about differential expression analysis and validation of candidate gene expression patterns using qRT-PCR, see Supplementary Methods 4 and Supplementary Table 3.

### Temperature and viral infection influence aphid lifespan and fecundity

We employed a full-factorial experimental design that tested the effects of co-occurrence (at two levels: co-occurrence and without co-occurrence), temperature (at six levels: 15, 18, 21, 23, 26, and 28 °C), and viral infection (at three levels: no virus, BYDV-PAV, and BYDV-RMV), on the lifespan (number of days alive after first nymph) and fecundity (number of offspring) of the two aphid species. This experiment was performed in controlled chambers (Conviron CMP 3244, 1.15 m × 2.5 m × 2 m) at a light intensity of 94.17 W m^−2^ and 64% RH. To start each trial, we placed one first-instar nymph (without co-occurrence treatments) or two first-instar nymphs, one from each aphid species (interspecific co-occurrence treatments), onto a plant as above. Each experimental unit was randomly located within the chamber. Each factor combination was replicated 15 times using new plants. The lifespan and fecundity were recorded daily and offsprings were removed daily using a paintbrush. After aphid lifespan and fecundity measurements were taken, the plants were frozen at −80 °C, and virus infection was confirmed using DAS-ELISA (as described above). Data were analyzed using a generalized linear model with Weibull distribution for lifespan data, and for fecundity we used Poisson family error because these data were counts^65^. All statistical analyses were conducted in JMP-Pro version 13.1.0 (SAS Institute 2018) and Python scipy library.

### Reporting summary

Further information on research design is available in the Nature Research Reporting Summary linked to this article.

## Supplementary information

Supplementary Information

Reporting Summary

## Data Availability

The source data underlying Figs. 1–5 and Supplementary Figs. 1–5 are provided as Supplementary Data and has been deposited on AEKOS data repository [10.25901/5d19bf3b1a81d]. The transcriptomic data generated from this study have been deposited in the genomic data in NCBI’s Sequence Read Archive and are accessible through the bioproject PRJNA314356.

## Source Data

Source Data
