# Supplementary material for: Enhanced heat tolerance of viral-infected aphids leads to niche expansion and reduced interspecific competition
Source: Nat Commun. 2020 Mar 4;11:1184. doi: 10.1038/s41467-020-14953-2 (PMC7055324; doi:10.1038/s41467-020-14953-2)
Supplement: Supplementary file 1 — Supplementary Information [file 41467_2020_14953_MOESM1_ESM.pdf]

Supplementary Information

Enhanced heat tolerance of virus-infected aphids leads to niche expansion and reduced interspecific competition

Porras et al.

Supplementary Methods

1. Lethal temperature 50 (LT_{50}) analysis

To measure the effects of the virus strains BYDV-PAV and BYDV-RMV on their insect vectors *Rhopalosiphum padi* and *R. maidis* we used a paired design, with two treatment groups: virus-free and viruliferous aphids (viruliferous *R. padi* BYDV-PAV, viruliferous *R. maidis* BYDV-RMV, virus-free *R. padi* and R. *maidis*). The experimental unit was an (4-day-old) adult aphid. We evaluated fifteen temperatures (18, 20, 22, 24, 26, 28, 30, 32, 34, 36, 38, 40, 42, 44, and 47 °C) for 56 min. Each treatment was replicated 10 times. Aphids were frozen at -80 °C, and virus infection was tested using DAS-ELISA. The data was analyzed using a logistic dose regression using Python's library *scipy* (version 3.6.0) following a logistic curve

$$y = \frac{1}{1+e^{\alpha+\beta x}}$$

Where α and β are parameters to be estimated, *y* is a binary outcome, equals 1 if an aphid is alive, 0 if it is dead and *x* is a temperature in Celcius. Estimates $\hat{\alpha}$ and $\hat{\beta}$ of parameters and α and β were obtained using Ordinary Least Square (OLS) method:

$$(\hat{\alpha}, \hat{\beta}) = arg_{\alpha,\beta} min + \sum_i \left(y_i - \frac{1}{1 + e^{\alpha+\beta e_i}}\right)$$

Minimization was performed using minimize routine *Broyden–Fletcher–Goldfarb–Shanno (*BFGS) and Nelder-Mead method to confirm the results. To calculate LT_{50}, $\hat{\alpha}$ and $\hat{\beta}$ were obtained on the previous step and solved the following equation for *x*:

$$\frac{1}{1 + e^{\hat{\alpha}+\hat{\beta}x}} = 0.5$$

We obtained the solution using the routine fsolve in Python (3.6.0)

2. Effects of viral infection on the locomotor capacity (walking speed) of aphids

The walking speed of virus-free and viruliferous aphids of each species was measured as in Gilchrist [1], by placing a single adult at the bottom of a test tube and knocking the tubes' bottom to elicit an escape response at six different environmental temperatures (14, 22, 26, 18, 30, and 35 °C) in a programmable walk-in chamber. We recorded the time it takes to walk the length of the tube (10 cm length) with a stopwatch. Aphids were frozen at -80 °C, and virus infection was tested using DAS-ELISA. Four treatment groups were evaluated as before: BYDV-PAV viruliferous *R. padi*, BYDV-RMV viruliferous *R. maidis*, and virus-free *R. padi* and *R. maidis*. Each treatment was replicated 100 times. We used a two-way ANOVA with temperature as a factor and the walking speed of the viruliferous and virus-free aphids as independent variable for each aphid species.

3. Thermal preference of aphids

We measured the effect of the virus strains on the thermal preference of their vectors following a paired design (virus-free and viruliferous aphid). For our artificial thermal gradient, we used an electric resistance (OMEGA Stamford, Connecticut, USA) and a thermoelectric cooler (Electro-Mechanical products, Inc., Lakewood, CO, USA) vertically placed surrounding the plant, both devices were connected to a DC power supply (VWR, Radnor, PA, USA) and automatically controlled using a computer interface (LabVIEW Student, National Instruments, Austin, TX, USA). An individual aphid was placed in the temperature-controlled surface and

after 30 minutes their body temperature was recorded using a thermocouple thermometer with RS232 output data logger (VWR, Radnor, PA, USA). After measurement the aphids were frozen at -80 °C, and virus infection was tested using DAS-ELISA.

4. Genes associated with heat thermal tolerance of viruliferous aphids

Differential expression analysis: The reads for each condition were trimmed and had library adapters removed using Trimmomatic v0.3 [2]. The reads were then aligned to the transcriptome built [nih.gov/bioprojec/ PRJNA314356] using Tophat [3]. Abundance of transcripts for each condition was quantified using Cufflinks [55], and transcript abundance for all conditions merged with cuffmerge. Transcript abundance was expressed in terms of Fragments Per Kilobase of exon model Mapped (FPKM). Differential expression analysis was followed with cuffdiff, using the quartile normalization method. The results of the differential analysis were then summarized using cummeRbund [4]. Significantly upregulated/downregulated genes were defined as those genes having an FDR < 0.05 and log2fold change > 2.0. To verify the presence/absence of virus in each sample, the short reads were blasted against the BYDV-PAV genome (GenBank NC_004750).

Validation of gene expression associated with thermal tolerance in *R. padi.* using quantitative real time PCR (qRT-PCR). Virus-free and viruliferous *R. padi* from the heat stress and held at room temperature conditions were obtained (20 aphids per sample, eight replicates per treatment). Aphids were flash-frozen in liquid nitrogen and stored at -80 ˚C. Next, we extracted RNA following the protocol above. The cDNA synthesis and quantitative real-time PCR were performed as in Fussnecker et al. [5]. Expression levels of candidate genes were

normalized to actin and ribosomal protein S8 (RPS8). The primer sequences are given in (Supplementary Table 2). Significant differences in expression levels among treatment groups were determined using *t*-test.

Supplementary Tables

Supplementary Table 1. Virus concentration in wheat leaves and viruliferous aphids used in experiments. Shown are the mean (± SE) for each virus concentration (ng).

Experiment	Treatment	Virus titer Mean	Virus titer SD
**Interspecific competition and infection by BYDV influences the distribution of aphids on thermally-heterogeneous plants**			
Plant surface temperature			
	**15 °C**		
	R. padi BYDV-PAV	619.72	120.23
	R. maidis BYDV-RMV	189.01	66.53
	**23°C**		
	R. padi BYDV-PAV	475.9	107.98
	R. maidis BYDV-RMV	156.51	47.45
	**28 °C**		
	R. padi BYDV-PAV	529.51	133.26
	R. maidis BYDV-RMV	237.85	105.07
Thermal preference of aphids in field			
	R. padi BYDV-PAV	775.26	94.90
	R. maidis BYDV-RMV	616.33	67.60
	BYDV-PAV-infected plants	659.52	90.53
	BYDV-RMV-infected plants	550.19	92.51
**Virus effects on thermal tolerance of aphids**			
CT_{Max} effect on offspring of virus-free vs. Viruliferous aphids			
	R. padi BYDV-PAV	579.3	
	R. maidis BYDV-RMV	518.83	
Lethal thermal dose			
	**18 °C**		

R. padi BYDV-PAV	516.14	65.22
R. maidis BYDV-RMV	481.96	67.44
**20°C**		
R. padi BYDV-PAV	537.69	60.16
R. maidis BYDV-RMV	498.21	43.69
**22°C**		
R. padi BYDV-PAV	511.29	96.76
R. maidis BYDV-RMV	488.3	61.52
**24°C**		
R. padi BYDV-PAV	541.37	36.6
R. maidis BYDV-RMV	512.05	33.91
**26°C**		
R. padi BYDV-PAV	549.57	59.24
R. maidis BYDV-RMV	487	41.69
**28°C**		
R. padi BYDV-PAV	547.74	34.65
R. maidis BYDV-RMV	514.64	45.93
**30°C**		
R. padi BYDV-PAV	564.03	37.73
R. maidis BYDV-RMV	491.33	43.45
**32°C**		
R. padi BYDV-PAV	545.94	37.95
R. maidis BYDV-RMV	542.85	69.13
**34°C**		
R. padi BYDV-PAV	542.2	48.17
R. maidis BYDV-RMV	511.07	26.7
**36°C**		
R. padi BYDV-PAV	536.17	47.07
R. maidis BYDV-RMV	512.32	60.84
**38°C**		
R. padi BYDV-PAV	507.27	110.18
R. maidis BYDV-RMV	522.05	35.38
**40°C**		
R. padi BYDV-PAV	535.13	49.28

	R. maidis BYDV-RMV	494.1	33.61
	**42°C**		
	R. padi BYDV-PAV	561.05	49.62
	R. maidis BYDV-RMV	478.75	42.63
	**44°C**		
	R. padi BYDV-PAV	560.77	49.23
	R. maidis BYDV-RMV	518.75	62.82
	**47°C**		
	R. padi BYDV-PAV	536.93	67.44
	R. maidis BYDV-RMV	513.21	35.92
Locomotor capacity			
	R. padi BYDV-PAV	480.52	86.44
	R. maidis BYDV-RMV	402.77	47.72
**Temperature and viral infection modulate interspecific competition between aphids**			
	**15 °C**		
	R. padi		
	BYDV-PAV infected plant	680.78	152.68
	BYDV-RMV infected plant	288.21	89.84
	R. maidis		
	BYDV-PAV infected plant	552.73	165.51
	BYDV-RMV infected plant	274.7	119.04
	Competition		
	BYDV-PAV infected plant	521.89	84.13
	BYDV-RMV infected plant	202.73	83.55
	**18 °C**		
	R. padi		
	BYDV-PAV infected plant	664.02	100.4
	BYDV-RMV infected plant	302.02	52.36
	R. maidis		
	BYDV-PAV infected plant	644.81	71.54
	BYDV-RMV infected plant	277.14	57.65
	Competition		

BYDV-PAV infected plant	523.68	82.07
BYDV-RMV infected plant	203.8	52.12
**21 °C**		
R. padi		
BYDV-PAV infected plant	675	142.03
BYDV-RMV infected plant	318.15	138.71
R. maidis		
BYDV-PAV infected plant	719.62	133.94
BYDV-RMV infected plant	353.33	104.02
Competition		
BYDV-PAV infected plant	614.9	101.25
BYDV-RMV infected plant	230.89	57.62
**23 °C**		
R. padi		
BYDV-PAV infected plant	708.1	162.18
BYDV-RMV infected plant	295.59	94.52
R. maidis		
BYDV-PAV infected plant	687.63	165.62
BYDV-RMV infected plant	265.47	83.84
Competition		
BYDV-PAV infected plant	608.42	96.36
BYDV-RMV infected plant	182.73	52.88
**26 °C**		
R. padi		
BYDV-PAV infected plant	737.96	154.83
BYDV-RMV infected plant	261.78	73.67
R. maidis		
BYDV-PAV infected plant	712.73	142.78
BYDV-RMV infected plant	269.88	81.35
Competition		
BYDV-PAV infected plant	582.91	124.77
BYDV-RMV infected plant	142.32	48.32
**28 °C**		
R. padi		

BYDV-PAV infected plant	704.61	133.3
BYDV-RMV infected plant	224.36	11.17
R. maidis		
BYDV-PAV infected plant	710.96	109.27
BYDV-RMV infected plant	202.16	73.48
Competition		
BYDV-PAV infected plant	491.71	125.72
BYDV-RMV infected plant	235.52	57.62

Supplementary Table 2. List of differentially expressed transcripts detected by RNA-Seq between viruliferous *R. padi* exposed to heat stress and virus-free unstressed individuals. Table is sorted by Log2Fold change in expression.

**B**	similar to HSP 70 (LOC100167145), mRNA	3.54723	6.72366	0.00005	0.0013897
**XM_001952245**	similar to CG13926 CG13926-PA (LOC100161962), mRNA	3.4708	7.87674	0.00005	0.0013897
**XM_001949446**	similar to SPRY domain-containing SOCS box protein 3 (SSB-3) (LOC100160157), mRNA	3.08102	7.74982	0.00005	0.0013897
**XM_001948236**	similar to kelch-like 10 (LOC100165373), mRNA	3.02197	3.72117	0.00005	0.0013897
**XM_001952245**	similar to CG13926 CG13926-PA (LOC100161962), mRNA	2.98099	3.65214	0.00005	0.0013897
**XM_001950531**	similar to cubilin (LOC100160169), partial mRNA	2.83574	2.86856	0.0001	0.00245653
**XM_001943573**	similar to conserved hypothetical protein (LOC100161897), mRNA	2.79958	7.92519	0.00005	0.0013897
**XM_001947729**	similar to AGAP010241-PA (LOC100159801), mRNA	2.77018	3.01805	0.0005	0.00870628
**XM_001946300**	similar to starvin CG32130-PE	2.38038	5.42831	0.00005	0.0013897

	(LOC100161093), mRNA				
**XM_001950029**	similar to HSP70 (LOC100163625), mRNA	2.05397	4.14144	0.00005	0.0013897
**A**	similar to HSP 70 (LOC100159543), mRNA	1.98636	4.17174	0.00005	0.0013897
**XM_001950295**	similar to AGAP001388-PA (LOC100167026), mRNA	1.83251	2.87004	0.00005	0.0013897
**XM_001952564**	similar to Pox meso CG9610-PC (LOC100164585), mRNA	1.81566	2.47368	0.00035	0.00665665
**XM_001946796**	similar to cuticle protein (LOC100164365), mRNA	1.81067	3.75782	0.00005	0.0013897
**XM_001952411**	hypothetical protein	1.77514	4.4022	0.00005	0.0013897
**XLOC_007285**	hypothetical protein	1.76018	3.58834	0.00005	0.0013897
**XM_001951757**	similar to Hsc70Cb CG6603-PA (LOC100163455), mRNA	1.66551	3.9663	0.00005	0.0013897
**XM_001946113**	similar to conserved hypothetical protein (LOC100160731), partial mRNA	1.64541	2.32984	0.00005	0.0013897
**XM_001950535**	hypothetical protein LOC100160667 (LOC100160667), mRNA	1.49369	3.36387	0.00005	0.0013897
**XM_001951174**	similar to phosphatidylethanolamine-binding protein (LOC100159619), mRNA	1.46651	3.62649	0.00005	0.0013897
**XM_001950602**	similar to Bm44 (LOC100164272), mRNA	1.4459	3.66428	0.00005	0.0013897

**XM_001944563**	similar to cuticular protein 124, RR-2 family (AGAP003390-PA) (LOC100159526), mRNA	1.43491	2.55522	0.00005	0.0013897
**XM_001949610**	similar to AGAP005961-PA (LOC100159739), mRNA	1.42503	3.97074	0.00005	0.0013897
**XM_001947269**	similar to carboxylesterase; esterase FE4 (LOC100162163), mRNA	1.41566	2.61986	0.00005	0.0013897
**XM_001945552**	similar to zinc finger, AN1-type domain 1 (LOC100161648), mRNA	1.39102	3.7426	0.00005	0.0013897
**XM_001948876**	hypothetical protein LOC100164120 (LOC100164120), mRNA	1.38419	4.2883	0.00005	0.0013897
**XM_001949026**	similar to HSP 40 (LOC100165140), mRNA	1.37772	3.44969	0.00005	0.0013897
**XLOC_002005**	hypothetical protein	1.35482	3.38934	0.00005	0.0013897
**XM_001949521**	similar to phosphatidylethanolamine-binding protein (LOC100159677), mRNA	1.32648	2.30789	0.00035	0.00665665
**XLOC_001719**	hypothetical protein	1.32576	1.94136	0.00105	0.0152641
**XM_001949793**	similar to zinc finger, AN1-type domain 1 (LOC100161565), mRNA	1.31656	3.44912	0.00005	0.0013897
**XM_001948483**	similar to AGAP006994-PA (LOC100164302), mRNA	1.30138	3.69292	0.00005	0.0013897
**XM_001947712**	similar to Collagen alpha-2(IV) chain	1.29928	2.57958	0.00005	0.0013897

	(LOC100161577), mRNA				
**C**	similar to HSP90, transcript variant 2 (LOC100168702), mRNA	1.29326	2.05397	0.00115	0.0162681
**XM_001949597**	similar to Bm44 (LOC100163495), mRNA	1.28714	3.66728	0.00005	0.0013897
**XM_001950782**	similar to EFHC1 (LOC100167091), mRNA	1.27855	2.02378	0.0011	0.0157619
**XM_001949396**	similar to CG14534 CG14534-PA (LOC100160127), mRNA	1.26251	2.86087	0.00005	0.0013897
**XM_001947210**	similar to WD40-repeat protein upregulated in HCC (LOC100161034), mRNA	1.22981	2.41092	0.0002	0.00430246
**XM_001949673**	similar to laminin A chain, putative (LOC100169395), partial mRNA	1.17774	3.06869	0.00005	0.0013897
**XLOC_001752**	hypothetical protein	1.16516	2.46479	0.0001	0.00245653
**XM_001951301**	similar to collagen alpha-2(IV) chain (LOC100163579), partial mRNA	1.15424	2.40324	0.00005	0.0013897
**XM_001943513**	similar to CG11474 CG11474-PA (LOC100168038), mRNA	1.15068	2.96903	0.00005	0.0013897
**XM_001947825**	similar to RR2 cuticle protein 2 (LOC100167758), mRNA	1.10469	2.52006	0.00005	0.0013897
**XM_001943052**	hypothetical protein LOC100166240 (LOC100166240), mRNA	-1.12652	-2.55293	0.00005	0.0013897

**XM_001946339**	similar to conserved hypothetical protein (LOC100167032), mRNA	-1.1306	-2.6724	0.00005	0.0013897
**XM_001952694**	similar to Bardet-Biedl syndrome 7 (LOC100163075), mRNA	-1.15844	-2.46801	0.00005	0.0013897
**XLOC_007266**	hypothetical protein	-1.18434	-1.97115	0.00105	0.0152641
**XM_001952469**	similar to conserved hypothetical protein (LOC100167324), mRNA	-1.18471	-2.93336	0.00005	0.0013897
**XM_001951381**	similar to CG15027 CG15027-PA (LOC100166086), mRNA	-1.20308	-2.57291	0.00005	0.0013897
**XM_001947823**	similar to AGAP003788-PA (LOC100164100), mRNA	-1.22999	-3.32765	0.00005	0.0013897
**XM_001952468**	similar to arrestin homolog (LOC100166316), mRNA	-1.23097	-3.33668	0.00005	0.0013897
**XLOC_002031**	hypothetical protein	-1.24641	-3.17159	0.00005	0.0013897
**XM_001951559**	similar to lipase (LOC100168142), mRNA	-1.25677	-2.76936	0.00005	0.0013897
**XM_001945056**	similar to AGAP009730-PA (LOC100159321), mRNA	-1.2803	-3.33188	0.00005	0.0013897

Supplementary Table 3. Primer sequences for validation of candidate gene expression patterns using

Gene	Description	Accession No.	Primer sequences (5'-3')	Length (bp)
A	*R. padi* heat shock protein 70-1 (hsp70-1)mRNA	KU311037.1	AGCCACTCAACCTGTCCATC	0.9786
			GGTGAACGTCTGGGTTTGTT	
B	*R. padi* heat shock protein 70-2 (hsp70-2) mRNA	KU311038.1	AGTAGAGGTCATCGCCAACG	0.9416
			CCGCAATCGTTTACCACTTT	
C	*R. padi* heat shock protein 90 (hsp90) mRNA	KR078260.1	GTTGACCGATCCATCCAAAT	0.9787
			GGCTACCAAATAGGCGGAGT	
Actin	House keeping		TGCCAACACTGTCCTTTCTG	0.8678
			AGAATTGACCCACCAATCC	
Ribosomal protein S8	House keeping	GAJW01000269	GTCGTCCGAGCCATTCTTT	0.9132
			TCCTGTCTTCCTGCGTTTATG	

Supplementary Table 4. Analysis of deviance (type II) tables of two-way ANOVAS from experiments testing the effects of viral infection (virus-free, BYDV-PAV, BYDV-RMV) and interspecific co-occurrence (with, without), on the height (cm) and temperature (°C) of the plant preferred by *Rhopalosiphum padi* and *R. maidis*.

A) Response: preferred temperature by *R. padi:*

$F_{3,236}$= 362.26, P<0.001

Model Effect:	d.f.	χ^2	P
virus	1	78.81	<0.0001
co-occur.	1	981.46	<0.0001
co-occur:virus	1	26.50	<0.0001

B) Response: preferred distance *R. padi:*

$F_{3,263}$= 6330.82, P<0.001

virus	1	792.43	<0.0001
co-occur	1	1327.66	<0.0001
co-occur:virus	1	63.25	<0.0001

C) Response: preferred temperature by *R. maidis*:

$F_{3,263}$= 1.22, P= 0.3005

virus	1	2.5189	0.1138
co-occur.	1	0.1356	0.7130
co-occur:virus	1	1.0273	0.3118

D) Response: preferred distance by *R. maidis*:

$F_{3,263}$= 2.85, P= 0.0378

virus	1	6.08	0.0143
.	1	1.78	0.1823
co-occur:virus	1	0.69	0.4052

Supplementary Table 5. Parameter estimates of analysis of deviance (type II) tables of two-way ANOVA from experiments testing the effects of viral infection (virus-free, BYDV-PAV, BYDV-RMV) and interspecific co-occurrence (with and without), on the preferred plant temperature and height of *Rhopalosiphum padi* and *R. maidis*.

Term	Estimate	Std Error	t Ratio	Prob>|t|
A) Response: Preferred temperature by *R. padi*				
Intercept	22.693875	0.10413	217.94	<.0001
virus[control]	-0.924458	0.10413	-8.88	<.0001
co-occur [padi]	-3.262208	0.10413	-31.33	<.0001
co-occur [padi]*virus[control]	0.536125	0.10413	5.15	<.0001
B) Response: preferred height by *R. padi*				
Intercept	18.40125	0.233496	78.81	<.0001
Infect[control]	-1.817083	0.233496	-7.78	<.0001
co-occur [padi]	-8.507917	0.233496	-36.44	<.0001
virus[control]* co-occur [padi]	1.8570833	0.233496	7.95	<.0001
C) Response: preferred temperature by *R. maidis*				
Intercept	19.267917	0.127853	150.70	<.0001
virus[control]	0.2029167	0.127853	1.59	0.1138
co-occur [maidis]	-0.047083	0.127853	-0.37	0.7130

Term	Estimate	Std Error	t Ratio	Prob>|t|
co-occur maidis]*Infect[control]	0.1295833	0.127853	1.01	0.3118

D) Response: preferred height by *R. maidis*

Intercept	9.3016667	0.287837	32.32	<.0001
virus[control]	0.71	0.287837	2.47	0.0143
co-occur [maidis]	-0.385	0.287837	-1.34	0.1823
co-occur [maidis]*virus[control]	0.24	0.287837	0.83	0.4052

Supplementary Table 6. Parameter estimates of analysis of deviance (type II) tables of full-factorial models from experiments testing the effects of viral infection (virus-free, BYDV-PAV, BYDV-RMV), temperature (15-28 °C), and co-occurrence (with and without), on the lifespan (days) and fecundity (# offspring) of *Rhopalosiphum maidis* and *R. padi* raised on wheat plants.

Term	Estimate	Std Error	Lower 95%	Upper 95%
A) Response: lifespan of *R. padi* $\chi 2$=600.70, DF =11, *P* <0.0001, AICc=2835.57				
Intercept	2.97	0.11	2.748	3.196
virus[BYDV-PAV]	0.04	0.02	-0.007	0.100
virus[BYDV-RMV]	0.03	0.02	-0.022	0.084
temp	-0.006	0.005	-0.016	0.003
virus[BYDV-PAV]*(temp-21.83)	0.01	0.007	-0.002	0.027
virus[BYDV-RMV]*(temp-21.83)	-0.01	0.007	-0.028	0.001
co-occur.[0]	-0.01	0.01	-0.048	0.023
virus[BYDV-PAV]* co-occur. [0]	-0.03	0.02	-0.088	0.018
virus[BYDV-RMV]* co-occur. [0]	-0.0007	0.02	-0.054	0.052
(temp-21.8333)* co-occur. [0]	-0.007	0.005	-0.017	0.002
virus[BYDV-PAV]*(temp-21.83)* co-occur. [0]	0.014	0.007	-0.0007	0.029

Term	Estimate	Std Error	Lower 95%	Upper 95%
virus[BYDV-RMV]*(temp-21.83)* co-occur. [0]	0.003	0.007	-0.011	0.018
δ	0.383	0.01	0.352	0.413

Term	Estimate	Std Error	L-R $\chi2$	Prob> $\chi2$	Lower CL	Upper CL
B) Response: fecundity of *R. padi* F=883.15, DF =11, P <0.0001, AICc=751.84						
Intercept	-0.12	0.17	0.51	0.4747	-0.460	0.20[illegible]
temp	0.12	0.006	378.81	<.0001*	0.114	0.14
virus[PAV]	0.47	0.03	138.66	<.0001*	0.395	0.55
virus[RMV]	0.20	0.04	24.47	<.0001*	0.124	0.28[illegible]
co-occur	0.04	0.03	2.15	0.1421	-0.015	0.10[illegible]
(temp-21.83):virus[PAV]	0.02	0.008	6.10	0.0135*	0.004	0.03[illegible]
(temp-21.83):virus[RMV]	-0.01	0.009	2.12	0.1453	-0.031	0.00[illegible]
(temp-21.83): co-occur	0.005	0.006	0.61	0.4317	-0.008	0.01[illegible]
Virus[PAV]: co-occur	0.01	0.03	0.18	0.6638	-0.060	0.09[illegible]
Virus[RMV]: co-occur	0.02	0.04	0.46	0.4936	-0.052	0.10[illegible]
Virus[PAV]: co-occur:(temp-21.83)	-0.006	0.008	0.51	0.4748	-0.023	0.01[illegible]
Virus[RMV]: co-occur:(temp-21.83)	-0.001	0.009	0.02	0.8718	-0.019	0.01[illegible]

Term	Estimate	Std Error	Lower 95%	Upper 95%

Term	Estimate	Std Error	Lower 95%	Upper 95%
A) Response: lifespan of *R. maidis* $\chi2$=31.86, DF =11, *P* <0.0001, AICc=3186.62				
Intercept	2.97	0.11	2.748	3.196
virus[BYDV-PAV]	0.04	0.02	-0.007	0.100
virus[BYDV-RMV]	0.03	0.02	-0.022	0.084
temp	-0.006	0.005	-0.016	0.003
virus[BYDV-PAV]*(temp-21.83)	0.01	0.007	-0.002	0.027
virus[BYDV-RMV]*(temp-21.83)	-0.01	0.007	-0.028	0.001
co-occur.[[0]	-0.01	0.01	-0.048	0.023
virus[BYDV-PAV]*co-occur.[0]	-0.03	0.02	-0.088	0.018
virus[BYDV-RMV]* co-occur.[[0]	-0.0007	0.02	-0.054	0.052
(temp-21.83)* co-occur.[[0]	-0.007	0.005	-0.017	0.002
virus[BYDV-PAV]*(temp-21.83)* co-occur.[[0]	0.01	0.007	-0.0007	0.029
virus[BYDV-RMV]*(temp-21.83)* co-occur.[[0]	0.003	0.007	-0.011	0.018
δ	0.38	0.01	0.352	0.413

Term	Estimate	Std Error	L-R $\chi2$	Prob> $\chi2$	Lower CL	Upper CL
D) Response: fecundity of *R. maidis* *F*= 182.79 DF=11, *P* <0.0001, AICc=688.67						
Intercept	2.92	0.176	236.88	<.0001*	2.581	3.271
temp	-0.02	0.008	11.49	0.0007*	-0.043	-0.011
virus[PAV]	0.56	0.04	151.33	<.0001*	0.467	0.644
virus[RMV]	-0.009	0.05	0.03	0.8452	-0.110	0.089
co-occur	-0.01	0.03	0.11	0.7342	-0.084	0.059
(temp-21.83): co-occur	-0.002	0.008	0.09	0.7592	-0.018	0.013
virus[PAV]: co-occur	-0.02	0.04	0.26	0.6065	-0.111	0.065
virus[RMV]: co-occur	0.06	0.05	1.41	0.2342	-0.039	0.160
virus[PAV]: co-occur:(temp-21.83)	-0.001	0.01	0.02	0.8706	-0.021	0.018
virus[RMV]: co-occur:(temp-21.83)	0.009	0.01	0.76	0.3825	-0.012	0.031
virus[PAV]:(temp-21.83)	0.01	0.01	3.05	0.0807	-0.002	0.037
virus[RMV]:(temp-21.83)	0.0008	0.01	0.005	0.9414	-0.021	0.022

Supplementary Table 7. Analysis of Deviance (type II) tables of full-factorial models from experiments testing the effects of viral infection (virus-free, BYDV-PAV, BYDV-RMV), temperature (15-28 °C), and co-occurrence (with, without), on the lifespan:fecundity ratio of *Rhopalosiphum padi* and *R. maidis* raised on wheat plants.

**Source**	**DF**	**L-R ChiSquare**	**Prob>ChiSq**
virus	2	21.209237	<.0001*
temp	1	1.6771834	0.1953
co-occur.	1	0.8607539	0.3535
virus*temp	2	1.6990189	0.4276
virus* co-occur.	2	0.3451097	0.8415
co-occur.*temp	1	0.3242202	0.5691
co-occur.*temp*virus	2	0.7349149	0.6925

Supplementary Table 8. Parameter estimates of analysis of deviance (type II) tables of full-factorial models from experiments testing the effects of viral infection (virus-free, BYDV-PAV, BYDV-RMV), temperature (15-28 °C), and co-occurrence (with and without), on the lifespan:fecundity ratio of *Rhopalosiphum maidis* and *R. padi* raised on wheat plants.

Term	Estimate	Std Error	L-R ChiSquare	Prob>ChiSq	Lower CL	Upper CL
Intercept	-0.332959	0.342623	0.9582425	0.3276	-1.015349	0.3291682
virus[BYDV-PAV]	0.3733607	0.0953122	14.553915	0.0001*	0.1843027	0.55858
virus[BYDV-RMV]	-0.001637	0.1048677	0.0002439	0.9875	-0.21272	0.1992789
temp	-0.020248	0.0156173	1.6771834	0.1953	-0.050863	0.0104245
co-occur. [0]	-0.064978	0.07011	0.8607539	0.3535	-0.203061	0.0722615
virus[BYDV-PAV]*(temp-21.83)	0.005053	0.0212584	0.0565035	0.8121	-0.036645	0.0467873
virus[BYDV-RMV]*(temp-21.83)	0.0221959	0.0234516	0.898057	0.3433	-0.023699	0.0684293
virus[BYDV-PAV]* co-occur. [0]	0.0474763	0.0953122	0.2480796	0.6184	-0.139667	0.2346902
virus[BYDV-RMV]* co-occur. [0]	-0.000308	0.1048677	8.6213e-6	0.9977	-0.207206	0.2052917
co-occur. [0]*(temp-21.8333)	-0.008889	0.0156173	0.3242202	0.5691	-0.039582	0.0217052

Term	Estimate	Std Error	L-R ChiSquare	Prob>ChiSq	Lower CL	Upper CL
co-occur. [0]*(temp-21.8333)*virus[BYDV-PAV]	-0.004909	0.0212584	0.0533275	0.8174	-0.046662	0.036771
co-occur. [0]*(temp-21.8333)*virus[BYDV-RMV]	-0.013331	0.0234516	0.3235869	0.5695	-0.059534	0.0325953

Supplementary Figures

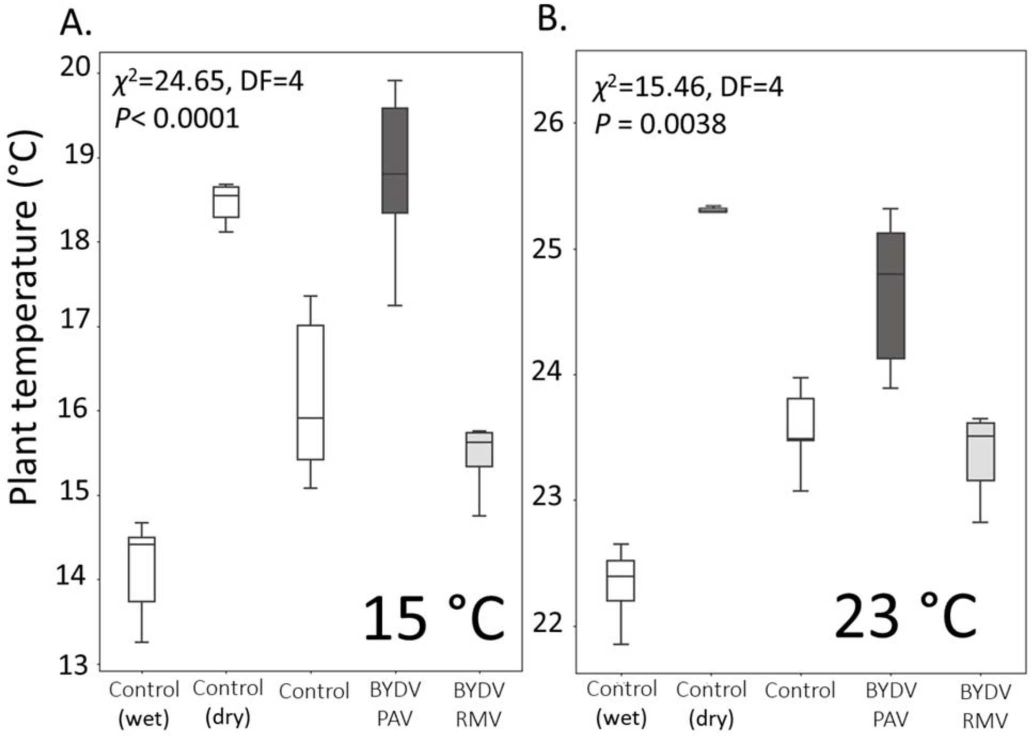

Supplementary Figure 1. Virus effects on plant temperature. We used infra-red (IR) thermal imaging to measure the effects of BYDV infection on plant surface temperature measurements in a climate-controlled chamber at 50% RH and two different environmental temperatures, 15°C **(a)** and 23°C **(b).** [Nonparametric ANOVAs, Kruskal-Wallis; boxplots display median line, interquartile range (IQR) boxes, 1.5*IQR whiskers; *n* = 6]. Centre line in box represents mean and whiskers show minimum to maximum. Source data are provided as a Source Data file.

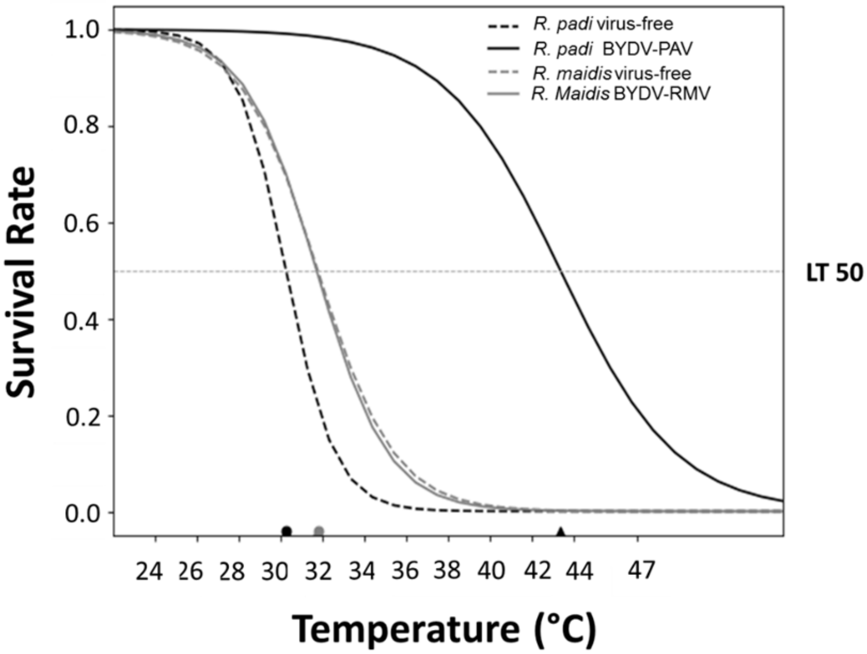

Supplementary Figure 2. Survival of *Rhopalosiphum padi* and *R. maidis* at 18, 20, 22, 24, 26, 28, 30, 32, 34, 36, 38, 40, 42, 44, and 47 °C, LT_{50} (temperature at which 50% of population dies -$LT_{50;}$ n = 50). Equations and coefficients for curves*:* virus-free *R. padi*: logit5ml[x]= 1/(1 + Exp[-25.68 + 0.84*x]; *R. padi* infected with BYDV-PAV: logit1ml[x] = 1/(1 + Exp[-15.64 + 0.36*x]); virus-free *R. maidis*: logit4ml[x]= 1/(1 + Exp[-17.29 + 0.54*x]); *R. maidis* infected with BYDV -RMV: logit3ml[x]= 1/(1 + Exp[-18.45 + 0.58*x]). Source data are provided as a Source Data file.

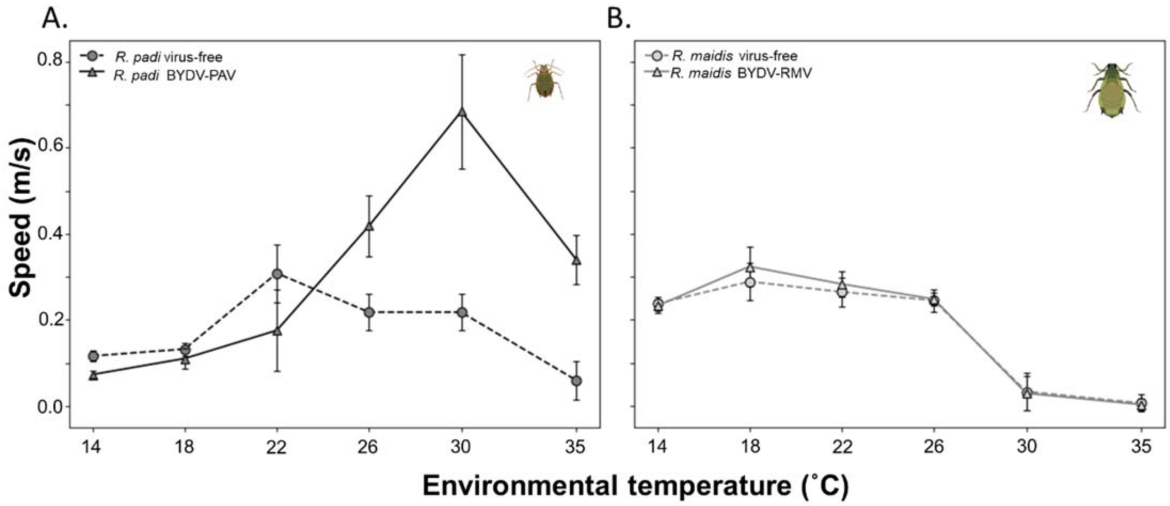

Supplementary Figure 3. Performance curves for virus-free and viruliferous *R. padi* and *R. maidis* at seven different temperatures. The curves describe the correlation of temperature and walking speed. Walking speed was higher in viruliferous *R. padi* at temperatures above 26 °C (two-way ANOVA *R. padi*: $F_{11,1188}$= 836.78, $P \leq 0.0001$; *R. maidis*: $F_{11,1188}$=1611.62, $P\leq$ 0.0001). Mean ± SE (n = 100 per treatment). Source data are provided as a Source data file.

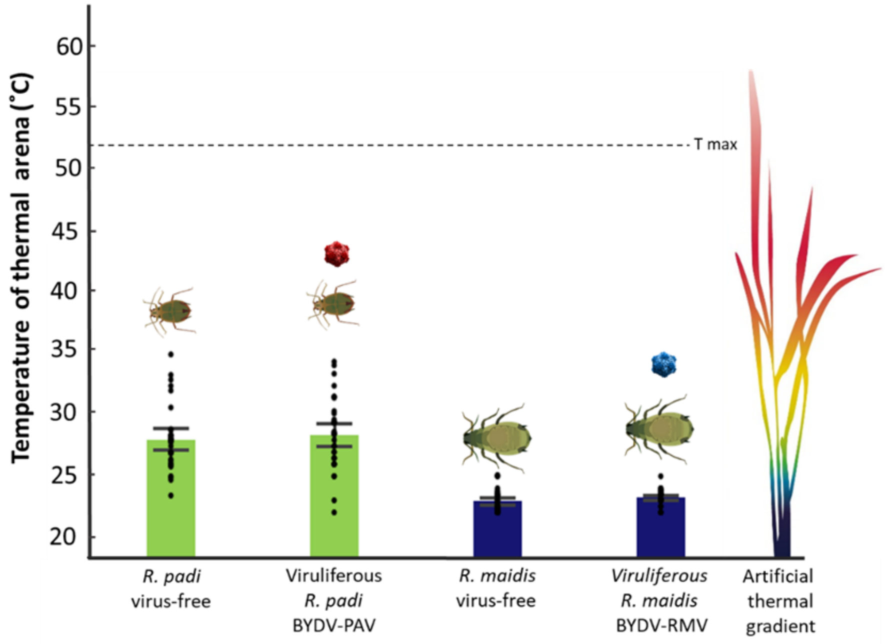

Supplementary Figure 4. Thermal preferences of virus-free and viruliferous *Rhopalosiphum padi* and *R. maidis* in an artificial thermal arena. Temperature was measured using IR thermal camera. Thermal scale (plant icon), bar colors indicate the temperature of the aphid's location. We characterized the thermal preference of each aphid species using a controlled thermal gradient in the host plant. *R. padi* selected areas with temperature between 23 to 26.5 °C, while *R. maidis* preferred temperatures between 20 to 23.3 °C. Virus infection did not significantly influence thermal preferences under these conditions [*R. padi*: Student's *t*-test = 0.60, *df*=57, *P* = 0.54; *R. maidis*: *t* = 1.79, *df*=57, *P* = 0.07]. Mean ± SE (*n* = 35 per treatment). Source data are provided as a Source data file.

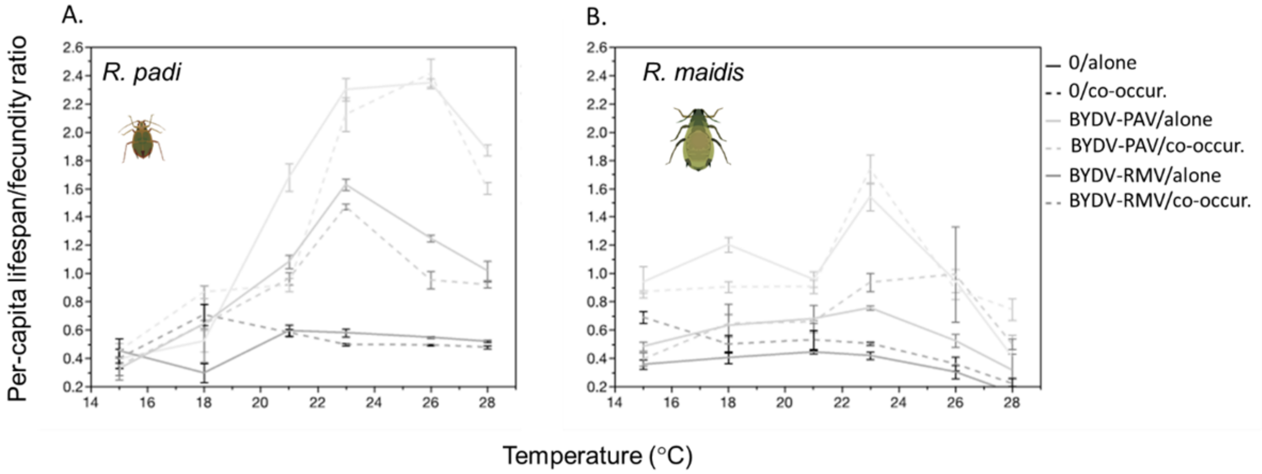

Supplementary Figure 5. Effects of viral infection on the per-capita lifespan:fecundity ratio of *R. padi* and *R. maidis* (occurring separately and together) across a range of environmental temperatures. Mean ± SE (*n* = 15 per treatment). Source data are provided as a Source data file.

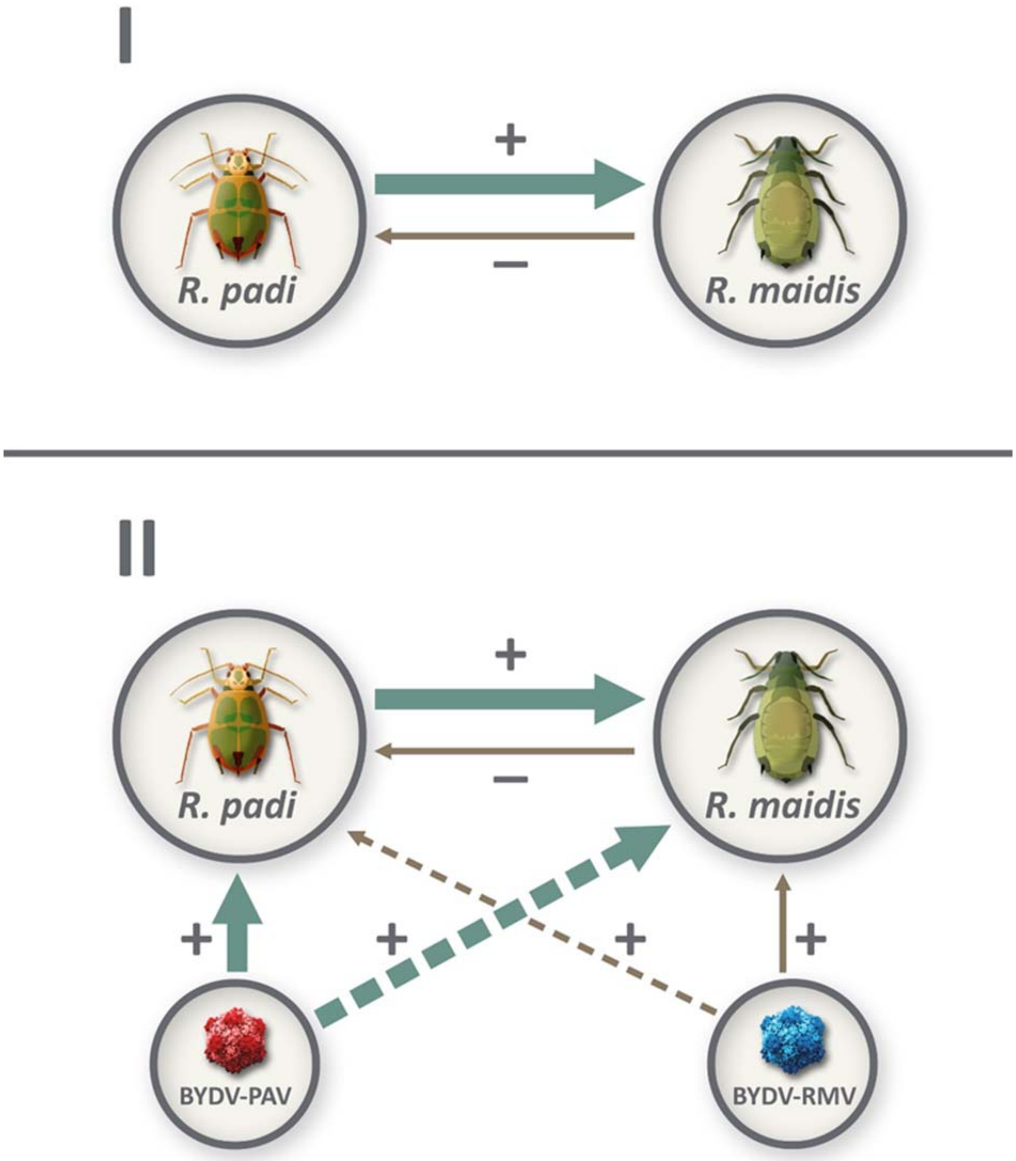

Supplementary Figure 6. Hypothesized interactions among aphid vectors and BYDV strains. Solid lines indicate direct effects, dashed lines represent indirect effects, and line weight shows the strength of positive (+) and negative (-) effects.

Supplementary References

1. Gilchrist, G. W. A quantitative genetic analysis of thermal sensitivity in the locomotor performance curve of *Aphidius ervi*. *Evolution* **50**, 1560—1572 (1996).
2. Bolger, A. M., Lohse, M., & Usadel, B. Trimmomatic: a flexible trimmer for Illumina sequence data. *Bioinformatics* **30**, 2114–2120 (2014).
3. Trapnell, C., Lior, P. & Steven, L. S. TopHat: discovering splice junctions with RNA-Seq. *Bioinformatics* **25**, 1105–1111 (2009).
4. Goff, L. A., Trapnell, C. & Kelley, D. CummeRbund: visualization and exploration of Cufflinks high-throughput sequencing data. R Package Version 2.2.0 (2012).
5. Fussnecker, B. L., McKenzie, A. M. & Grozinger, C. M. cGMP modulates responses to queen mandibular pheromone in worker honey bees. *J. Comp. Physiol. A Neuroethol. Sens. Neural Behav. Physiol.* **197**, 939–948 (2011).
