# Supplementary material for: Enhanced heat tolerance of viral-infected aphids leads to niche expansion and reduced interspecific competition
Source: Nat Commun. 2020 Mar 4;11:1184. doi: 10.1038/s41467-020-14953-2 (PMC7055324; doi:10.1038/s41467-020-14953-2)
Supplement: Supplementary file 2 — Reporting Summary [file 41467_2020_14953_MOESM2_ESM.pdf]

nature research

Corresponding author(s): NCOMMS-19-20056A

Last updated by author(s): 2/6/2020

Reporting Summary

Nature Research wishes to improve the reproducibility of the work that we publish. This form provides structure for consistency and transparency in reporting. For further information on Nature Research policies, see Authors & Referees and the Editorial Policy Checklist.

Statistics

For all statistical analyses, confirm that the following items are present in the figure legend, table legend, main text, or Methods section.

n/a	Confirmed	
☐	☒	The exact sample size (n) for each experimental group/condition, given as a discrete number and unit of measurement
☐	☒	A statement on whether measurements were taken from distinct samples or whether the same sample was measured repeatedly
☐	☒	The statistical test(s) used AND whether they are one- or two-sided<br>*Only common tests should be described solely by name; describe more complex techniques in the Methods section.*
☒	☐	A description of all covariates tested
☐	☒	A description of any assumptions or corrections, such as tests of normality and adjustment for multiple comparisons
☐	☒	A full description of the statistical parameters including central tendency (e.g. means) or other basic estimates (e.g. regression coefficient) AND variation (e.g. standard deviation) or associated estimates of uncertainty (e.g. confidence intervals)
☐	☒	For null hypothesis testing, the test statistic (e.g. F, t, r) with confidence intervals, effect sizes, degrees of freedom and P value noted<br>*Give P values as exact values whenever suitable.*
☒	☐	For Bayesian analysis, information on the choice of priors and Markov chain Monte Carlo settings
☐	☒	For hierarchical and complex designs, identification of the appropriate level for tests and full reporting of outcomes
☒	☐	Estimates of effect sizes (e.g. Cohen's d, Pearson's r), indicating how they were calculated

Our web collection on statistics for biologists contains articles on many of the points above.

Software and code

Policy information about availability of computer code

Data collection	FLIR tools software was used to take infrared pictures of aphid host plants (wheat) in the field and virus-infected host plants under laboratory conditions. LabVIEW 6i student software was used to control a flexible resistance connected to a DC power and a laptop.
Data analysis	JMP Pro 14 and Python 3.6.0 were used to analyse our data.

For manuscripts utilizing custom algorithms or software that are central to the research but not yet described in published literature, software must be made available to editors/reviewers. We strongly encourage code deposition in a community repository (e.g. GitHub). See the Nature Research guidelines for submitting code & software for further information.

Data

Policy information about availability of data

All manuscripts must include a data availability statement. This statement should provide the following information, where applicable:

- Accession codes, unique identifiers, or web links for publicly available datasets
- A list of figures that have associated raw data
- A description of any restrictions on data availability

The source data underlying Figs. 1-5, and Supplementary Figs 1-5 are provided as Supplementary Data and has been deposited on AEKOS data repository [https://doi.org/10.25901/5d19bf3b1a81d]. The transcriptomic data generated from this study have been deposited in the genomic data in NCBI’s Sequence Read Archive and are accessible through the bioproject PRJNA314356.

Field-specific reporting

Please select the one below that is the best fit for your research. If you are not sure, read the appropriate sections before making your selection.

☐ Life sciences ☐ Behavioural & social sciences ☒ Ecological, evolutionary & environmental sciences

For a reference copy of the document with all sections, see nature.com/documents/nr-reporting-summary-flat.pdf

Ecological, evolutionary & environmental sciences study design

All studies must disclose on these points even when the disclosure is negative.

Study description

-Virus effects on plant temperature-Thermal characterization of virus-free wheat plants under field conditions: We used infra-red (IR) thermal imaging to characterize the thermal profiles of the wheat plants in the field. We compared the temperature differences between stems and apical leaves of wheat plants. Experimental unit: a wheat plant (five-week old), n=20.

-Virus effects on plant temperature-Thermal characterization of virus-free and virus-infected plants under controlled conditions: We tested the effects of virus infection on host plant temperature. These experiments employed a full-factorial design to assess effects of viral infection (at three levels: virus-free plants, BYDV-PAV infected plants, and BYDV-RMV infected plants) and temperature (at three levels: 15, 23, and 28 °C). Experimental unit: wheat plant, each factor combination was replicated on six different plants. n= 6.

-Interspecific competition and BYDV-PAV infection influence the spatial distribution of R. padi: To examine how the distribution of aphids on a plant (microhabitat choices) was affected by interspecific competition and viral infection we conducted a field experiment using a raised bed with natural soil (120 cm x 100 cm x 40 cm, 50 cm above ground) at the Pennsylvania State University Horticultural Facility (University Park, PA). Wheat seeds were planted at 1 seed per 4.5 cm2 density. We used a 2 x 2 full-factorial experiment to test for the effects of BYDV (with and without) and aphid co-occurrence (one or two species) on the distribution of R. padi and R. maidis on host plants. Each factor combination was replicated ten times using randomly selected plants and aphid individuals (n= 10). For treatments using a single aphid species, we placed six adult aphids on the stem of a wheat plant. For interspecific co-occurrence treatments, we placed twelve aphids on the stem of each plant, six from each species as in the single-species treatment. All aphids were placed on plant stems using a paintbrush at a distance of 6 cm from the soil surface. Plants were enclosed in a transparent acrylic tube (4.5 cm diameter x 35 cm length) that had two windows (3 cm x 5 cm) covered with Lumite fabric (OHCO, Georgia, GA, USA). After 24 h we examined the distribution of the aphids on the plant and measured (i) the plant surface temperature at each aphid's location using a thermocouple thermometer with RS232 output data logger (VWR, Radnor, PA, USA) and (ii) the vertical distance from each individual to the ground. We removed the acrylic tube, measured the plant temperature and collected the aphids (the trial reading took 4h, starting at 9:00 am). Then, the viral infection of plants and aphids was confirmed using DAS-ELISA.

-Virus effects on thermal tolerance of aphids: Identification of the upper thermal limit (CTMax) of virus-free and viruliferous aphids: To determine (CTMax) for virus-free and viruliferous aphids of each species we employed a protocol modified from that of Ribeiro et al. (60) using a hotplate pelt with a programmable heating rate controlled by a computer interface (Sable Systems, LV, USA). The temperature was monitored by two independent thermocouple channels connected to a TC2000 Thermocouple Meter (Sable Systems). One thermocouple was attached to the surface of the hotplate, and the other sensor was attached inside of the metal container (aluminum clear glass top container 3.2 cm diameter x 2cm height, Eslinger & Co. Inc., MN, USA) in which we placed an individual aphid. This equipment was located inside an automated thermal chamber (dimensions of incubator's cabin: width 40.5 cm x 35 cm length x 40 cm height). We transferred an individual aphid (4-day old; aphids were grown on 20 different plants per infection treatment) onto the metal pelt and exposed it to increasing temperature at a rate of 0.1°C/minute until its locomotion stopped. CTMax was recorded when the aphid turned upside down and could no longer return to the upright position within 5 seconds. The aphid was returned to a plant for recovery. Data points were only considered valid if the aphid displayed normal activity two hours after a CTMax test. The aphid was then immediately frozen in liquid nitrogen and stored at -80 ̊C. Aphid infection status was then confirmed using DAS-ELISA.

-Changes in gene expression associated with thermal tolerance in R. padi: We explored potential molecular mechanisms underlying the increased heat tolerance of R. padi infected with BYDV-PAV) We extracted mRNA from virus-free and viruliferous R. padi maintained at room temperature (23 °C) and under heat stress (at CTMax). We then constructed transcriptomes for each treatment using Illumina Seq. and compared them to identify genes that were differentially regulated in viruliferous and virus-free aphids. We used a paired design to measure the differences in gene expression of virus-free and viruliferous R. padi exposed to thermal stress (at CTMax). As factors, we had two groups: virus infection (virus-free padi and viruliferous aphids), and temperature [room temperature (23 °C) and heat stress (CTMax)], with three replicates per treatment factor. An experimental unit consisted of 20 (4-day-old) adult aphids, which were collected from different host plants (15 plants per virus treatment).
II) qRT-PCR was used to quantify the expression levels of candidate genes identified from the transcriptomic analysis:We performed another trial of the CTMax experiment using a paired design to measure the differences in gene expression of virus-free and viruliferous R. padi exposed to thermal stress (at CTMax). As factors, we had two groups: virus infection (virus-free padi and viruliferous aphids), and temperature [room temperature (23 °C) and heat stress (CTMax)], with three replicates per combination of factors. A replicate trial consisted of 20 (4-day-old) adult aphids, which were collected from different host plants (15 plants per virus treatment), eight replicates per treatment factor.

-Temperature and virus infection influence aphid lifespan and fecundity: To identify effects of temperature, competition, and viral infection on R. padi and R. maidis (lifespan and fecundity), we employed a full-factorial experimental design that tested the effects of interspecific co-occurrence (at two levels: co-occurrence and without co-occurrence), temperature (at six levels: 15, 18, 21, 23, 26, and 28 °C), and viral infection (at three levels: no virus, BYDV-PAV, and BYDV-RMV), on the lifespan (number of days alive after first-

nymph) and fecundity (number of offspring) of the two aphid species. The experimental unit was an aphid, each treatment factor was replicated 15 times.

-Supplementary information -Effects of viral infection on the lethal thermal dose 50: We measured the effects of the virus strains BYDV-PAV and BYDV-RMV on their insect vectors Rhopalosiphum padi and R. maidis, respectively. We used a paired design, with two treatment groups: virus-free and viruliferous aphids (viruliferous R. padi BYDV-PAV, viruliferous R. maidis BYDV-RMV, virus-free R. padi, and R. maidis). The experimental unit was an (4-day-old) adult aphid. We evaluated fifteen temperatures (18, 20, 22, 24, 26, 28, 30, 32, 34, 36, 38, 40, 42, 44, and 47 °C) for 56 min. Each treatment was replicated 10 times.

-Supplementary information- Effects of viral infection on the locomotor capacity: We measured the walking speed of virus-free and viruliferous aphids of each species. We used a paired design, with two treatment groups: virus-free and viruliferous aphids (BYDV-PAV viruliferous R. padi, BYDV-RMV viruliferous R. maidis, and virus-free R. padi and R. maidis). The experimental unit was an adult (4-day-old) aphid, each treatment factor was replicated 100 times.

-Supplementary information- Behavioral thermal preference of aphids in an artificial thermal arena: We measured the effect of the virus strains (BYDV-PAV and BYDV-RMV) on the thermal preference of their vectors following a paired design, the treatments were a virus-free and viral infection. The experimental unit was an adult(4-day-old), each treatment factor was replicated 35 times.

Research sample

-Virus effects on plant temperature-Thermal profile of host plants: Wheat plants (Spring wheat cultivar, 5-weeks old) in a cultivated field. In nature, R. padi and R. padi live on grasses such as wheat plants. Wheat is a widely grown crop in Pennsylvania. Plants used in our experiment were grown using standard agronomic practices in the natural microclimatic conditions where the aphids live. Five-week old plants had completely developed a flag leave, so we could identify the stem from the flag leaves.

-Virus effects on plant temperature -Thermal characterization of virus-free and virus-infected plants under controlled conditions: We used wheat (Spring organic wheat) because it is the most common host plant of R. padi and R. maidis, and it is the main host of barley yellow dwarf virus. We studied the effects of barley yellow dwarf virus strain BYDV-PAV and BYDV-RMV because these strains are species-specific in our aphid species vectors, R. padi transmits BYDV-PAV while R. maidis transmits BYDV-RMV, being BYDV-PAV the most prevalent in nature. Virus strains BYDV-PAV and BYDV-RMV were also provided by Dr. S. Gray. Aphids were experimentally infected by allowing them to feed for three days on black oat leaves (Avena strigosa). Plants were infected by moving viruliferous aphids to healthy wheat plants and allowing them to feed for 12 days. The aphids were then removed, and virus infection was confirmed on each plant using a double-antibody sandwich, enzyme-linked immunosorbent assay (DAS-ELISA) (Agdia Inc., Elkhart, IN, USA). Absorbance was measured at 30 min intervals using a micro-titer plate reader-spectrophotometer (Spectramax 190, Molecular Devices, Silicon Valley, CA) with Agdia positive and negative controls on each plate. Samples with absorbance values (A405) three times higher than those of healthy plants were considered positive for virus infection. Virus titer for infected plants and aphids were estimated from the ELISA absorbance values following the protocol in Jiménez-Martínez et al. that allowed us to convert the absorbance into a virus titer concentration using a standard curve of known concentrations of purified virus (50, 100, 500 and 1000 ng), healthy plant-sap, and BYDV-infected plant.

-Interspecific competition and viral infection influence the spatial distribution of aphids: For a single no-competition trial, six adult aphids of one species (either R. padi or R. maidis) were placed on each plant. We used 4-day old adults to minimize interspecific competition and alarm pheromone. For competition trials, we placed twelve aphids (4-day old), six from each species, on each plant. This aphid density was still less than the density limits for aphid alarm pheromone release.

-Virus effects on thermal tolerance of aphids: Because BYDV affected the thermal biology of the host plant, we explored whether BYDV altered the thermal biology of its vectors. We infected individuals of R. padi and R. maidis with BYDV-PAV and BYV-RMV, respectively. The virus strain transmission is species-specific, therefore R. padi only transmits BYDV-PAV and R. maidis transmits BYDV-RMV. Aphids were experimentally infected by allowing them to feed for three days on black oat leaves (Avena strigosa) infected with the appropriate virus strain. We started new small colonies with these infected adults and used their offspring (4-day old adults) for our experiments). We measured the CTmax or upper thermal limit because it is the best proxy for aphid response to temperature fluctuations experienced in the field.

-Changes in gene expression associated with thermal tolerance in R. padi: the potential molecular mechanisms underlying the increased heat tolerance of R. padi infected with BYDV-PAV were explored by I) extracting mRNA from virus-free and viruliferous aphids. For each sample trial, we used 20 adults (4-day old) collected from different host plants (15 plants per virus treatment). This approach allowed us to validate that the observed heat tolerance is a general pattern in virus-infected R. padi. We used 20 adults because we needed to obtain a good amount of RNA for transcriptomic analyses. II) To quantify the expression levels of candidate genes identified from the transcriptomic analysis we used the same sample as described above (I) to keep consistency with the previous part of our experiment. We extracted RNA and synthesized cDNA to conduct qRT-PCR.

-Temperature and viral infection modulate interspecific competition between aphids: Our sample unit was an aphid that we followed from the first instar to death. We measured lifespan (number of days alive after the first instar) and fecundity (number of offspring) for each aphid species under no competition and competition. Lifespan allowed us to identify the optimal thermal threshold for each aphid species, and fecundity is a direct measurement for fitness.

-Supplementary information-Effects of viral infection on the lethal thermal dose 50: We measured the lethal thermal dose of virus-free and viruliferous aphids using (4-day-old) adult aphids (BYDV-PAV R. padi, BYDV-RMV R. maidis, and virus-free R. padi and R. maidis). These measurements validated our results from the experiment on virus effects on the upper thermal limit of vectors. This empirical information directly links locomotor performance to fitness and allows us to make inferences on how locomotor performance varies with viral infection in vectors.

-Supplementary information-Effects of viral infection on the locomotor capacity: We measured the walking speed of virus-free and viruliferous aphids of each species, we used (4-day-old) adult aphids (BYDV-PAV R. padi, BYDV-RMV R. maidis, and virus-free R. padi and R. maidis). These measurements validated and confirm our results of the experiment on virus effects on the upper thermal limit of vectors.

-Supplementary information- Behavioral thermal preference of aphids in an artificial arena: We assessed the effect of virus strain on the thermal preference of their vectors using (4-day-old) adult aphids (BYDV-PAV R. padi, BYDV-RMV R. maidis, and virus-free R. padi and R. maidis). These measurements allowed us to measure whether the virus affects the vector's behavior, specifically thermal preference.

Sampling strategy

-Virus effects on plant temperature-Thermal profile of host plants: We randomly selected twenty plants in a wheat field (5-week old). Taking infrared measurements on twenty plants allowed us to identify a temperature pattern. Our measurements were taken at the same environmental and lighting conditions using infra-red thermography.

-Virus effects on plant temperature - Thermal characterization of virus-free and virus-infected plants under controlled conditions: we randomly selected six plants (4- to 5-weeks old) from sets of 30 virus-free or virus-infected (BYDV-PAV or BYDV-RMV) plants. Then, the plants were acclimated to each temperature treatment (at three levels: 15, 23, and 28 °C) for 24 h in a controlled climate chamber (Conviron CMP 3244, 1.15 m x 2.5 m x 2 m) at a light intensity of 94.17 Wm-2 and 50% RH. This number of plants allowed us to identify temperature patterns. Infrared thermography was used to measure the temperature of each plant.

-Interspecific competition and viral infection influence the spatial distribution of aphids: The distribution of aphids on a wheat plant (microhabitat choices) as affected by interspecific co-occurrence and viral infection was examined by field experiments using a raised bed with natural soil (120 cm x 100 cm x 40 cm, 50 cm above ground). Wheat seeds were planted at 1 seed per 4.5 cm2 density. For treatments using a single aphid species, six adult aphids of one species (either R. padi or R. maidis) were placed on each plant. For interspecific co-occurrence treatments, we placed twelve aphids, six from each species, on each plant. All aphids were placed on the plant stem about six centimeters from the soil surface using a paintbrush, then each plant was caged within a transparent acrylic tube (4.5 cm diameter x 35 cm length) having two windows (3 cm x 5 cm) made of Lumite fabric (OHCO, Georgia, GA, USA). After 24 h we examined the distribution of the aphids on the plant measuring the distance from the soil surface to the location of each aphid on the host plant. We determined that aphids from parthenogenic laboratory colonies have a similar response, generally, five replicates showed a pattern, therefore using 10 replicates per treatment increased our chances of noticing any pattern of aphid distribution on the host plant.

-Virus effects on thermal tolerance of aphids: we employed a protocol modified from that of Ribeiro et al. 60, using a hotplate pelt with a programmable heating rate controlled by a computer interface (Sable Systems, LV, USA). The temperature was monitored by two independent thermocouple channels connected to a TC2000 Thermocouple Meter (Sable Systems) 53. One thermocouple was attached to the surface of the hotplate, and the other sensor was attached inside of the metal container (aluminum clear glass top container 3.2 cm diameter x 2cm height, Eslinger & Co. Inc., MN, USA) in which we placed an individual aphid. This equipment was located inside an automated thermal chamber (dimensions of incubator's cabin: width 40.5 cm x 35 cm length x 40 cm height). We transferred an individual aphid (4-day old; aphids were grown on 20 different plants per infection treatment) onto the metal pelt and exposed it to an increasing temperature at a rate of 0.1°C/minute until its locomotion stopped. CTMax was recorded when the aphid turned upside down and could no longer return to the upright position within 5 seconds. The aphid was returned to a plant for recovery. Data points were only considered valid if the aphid displayed normal activity two hours after a CTMax test 53. The aphid was then immediately frozen in liquid nitrogen and stored at -80 ̊C. Aphid infection status was then confirmed using DAS-ELISA.

-Changes in gene expression associated with thermal tolerance in R. padi: The potential molecular mechanisms underlying the increased heat tolerance of R. padi infected with BYDV-PAV, I) was explored by extracting mRNA from virus-free and viruliferous R. padi exposed to room temperature (23 °C) and heat stress (CTMax). In each of three replicates per treatment twenty adults (4-day old) provided an adequate amount of sample for Illumina HiSeq. 2500. R. padi is a small aphid (1.5-2.5mm), so it is necessary to ensure a good amount and high-quality RNA for sequencing.
(II) Quantification of expression levels of candidate genes identified from the transcriptomic analysis: We followed the same procedure as described above (I) to keep consistency with the previous part of the experiment. We extracted RNA and synthesized cDNA to conduct qRT-PCR. There were eight replicates per treatment. We compared the treatments against two housekeeping genes. We examined the results to determine changes in heat shock proteins.

-Temperature and viral infection modulate interspecific competition between aphids: We infected 180 plants with BYDV-PAV or BYDV-RMV [two trays with 90 plants in cones (1 plant/cone)]. We then randomly chose 45 plants from each virus-infected group of plants and control (virus-free) plants. Using a paint brush, we placed one first-instar nymph (without co-occurrence), or two first-instar nymphs, one from each aphid species (interspecific co-occurrence), onto a plant. Each plant was caged within a transparent acrylic tube (4.5 cm diameter x 35 cm length), having two windows (3 cm x 5 cm) made of Lumite fabric (OHCO, Georgia, GA, USA). Each experimental unit (aphid + plant) was randomly located within six controlled atmosphere chambers (Conviron CMP 3244, 1.15 m x 2.5 m x 2 m) set at different temperatures 15, 18, 21, 23, 26, and 28 °C), at a light intensity of 94.17 Wm-2 and 64 % RH. We recorded lifespan and fecundity per individual. Each factor-combination was replicated 15 times using new plants. R. padi and R. maidis colonies are parthenogenetic, which makes the sample variation small; therefore 15 replicates provided robust information about the performance of the aphid species at each combination of factors.

-Supplementary information-Effects of viral infection on the lethal thermal dose 50: An individual adult (4-day old; aphids were grown on 20 different plants per infection treatment) Each individual aphid was randomly chosen and gently manipulated using a paint brush to ensure its survival, and was placed on a metal pelt adapted to a ceramic hotplate inside automated thermal chamber (dimensions of incubator's cabin: width 40.5 cm x 35 cm length x 40 cm height, Sable System, LV, USA) and exposed to fifteen temperatures (18, 20, 22, 24, 26, 28, 30, 32, 34, 36, 38, 40, 42, 44, and 47 °C) for 56 min. We observed whether the aphid was alive or dead. Each treatment was replicated 10 times.

-Supplementary Information-Effects of viral infection on the locomotor capacity: We measured the walking speed of virus-free and viruliferous aphids of each species using (4-day-old) adult aphids (BYDV-PAV R. padi, BYDV-RMV R. maidis, and virus-free R. padi and R. maidis). We used a method modified from Gilchrist, by placing a single adult at the bottom of a test tube and knocking the tubes' bottom to elicit an escape response at six different environmental temperatures (14, 22, 26, 18, 30, and 35°C) in a programmable walk-in chamber. With a stopwatch we recorded the time it took for the aphid to walk the length of the tube (10 cm length). Each treatment was replicated 100 times; this sample size provides robust information on the performance of vectors.

-Supplementary information- Behavioral thermal preference of aphids in an artificial arena: We measured the effect of the virus strains on the thermal preference of their vectors using (4-day-old) adult aphids (BYDV-PAV R. padi, BYDV-RMV R. maidis, and virus-free R. padi and R. maidis). For our artificial thermal gradient, we used a flexible resistance (OMEGA Stamford, Connecticut, USA) and a thermoelectric cooling plate (Peltier Cell, Electro-Mechanical products, Inc., Lakewood, CO, USA) vertically placed surrounding the plant, both devices were connected to a DC power supply (VWR, Radnor, PA, USA) and automatically controlled using a computer interface (LabVIEW Student, National Instruments, Austin, TX, USA). An individual aphid was placed on the temperature-controlled surface, and after 30 minutes, their body temperature was recorded using a thermocouple thermometer with RS232 output data logger (VWR, Radnor, PA, USA). These measurements allowed us to measure whether the virus affects the vector's behavior, specifically thermal preference in an artificial thermal gradient. There were 35 replicates for each treatment factor.

Data collection

-Virus effects on plant temperature-Thermal profile of host plants: We characterized the natural temperature profiles of wheat plants in field using an IR thermal camera (T650SC; FLIR Inc., Wilsonville, OR, USA) with a 25 mm lens (15° field of view) positioned perpendicularly 1 m from the plant (three pictures per plant) from 15:00 to 15:30 h, solar elevation 60.5°. We obtained measurements of the temperature of the stem and flag leaf from the IR image as follows: In the IR image, we traced a vertical line in the middle of the plant from the base of the stem to the apical part of the flag leaf, using ResearchIR software (FLIR systems, USA). We then extracted twelve temperature data points, six on the stem and six on the flag leaf, from the middle of each plant. Temperature points were located ten pixels apart (1.0 cm). Measurements were taken by Mitzy Porras & Edwin Rajotte.

-Virus effects on plant temperature - Thermal characterization of virus-free and virus-infected plants under controlled conditions: We tested the effects of virus infection on plant temperatures in a controlled climate chamber, the temperature treatment had three levels: 15, 23, and 28 °C. We used a light intensity of 94.17 Wm-2 and 50% RH. Wheat plants were acclimated to each treatment for 24 h. Thermal images were obtained between 9:00 and 11:00am, using the IR Thermal Camera as above. Imaging protocols were modified from Leinonen and Jones 52 and included a plant sprayed with water (wet control) and a plant covered with Vaseline to inhibit transpiration (dry control) for each image. Temperature measurements were obtained similarly to previous experiments, but in this case, for 15 points (five each at the base of the stem, second tiller, and flag leaf). We used the average temperature of these points in our analysis. Measurements were taken by Mitzy Porras, Edwin Rajotte, Consuelo M. De Moraes, Mark C. Mescher. Guidance: Sylvain Pincebourde & James Marden.

-Interspecific competition and viral infection influence the spatial distribution of aphids: We examined the effects of viral infection and competition on the distribution of aphids on a plant (microhabitat choices). After 24 h of establishing our experimental set-up, we measured (i) the plant surface temperature at each aphid's location using a thermocouple thermometer with RS232 output data logger (VWR, Radnor, PA, USA) and (ii) the vertical distance from each individual to the soil surface. We removed the acrylic tube and measure the plant temperature and collected the aphids (the trial reading took 4h, starting at 9:00 am). Measurements were taken by Mitzy Porras, Edwin Rajotte & D. Smilnak.

-Virus effects on thermal tolerance of aphids: Individual aphids (4-day old; aphids were grown on 20 different plants per infection treatment) were placed on a metal pelt adapted to a ceramic hotplate inside an automated thermal chamber (dimensions of incubator's cabin: width 40.5 cm x 35 cm length x 40 cm height, Sable System, LV, USA) and exposed to increasing temperature at 0.1°C/minute until its locomotion stopped. We returned the aphids to a wheat plant and allowed them to recover for three hours. Only after we confirmed normal behavior, was the result judged valid. Measurements were taken by Mitzy Porras, Carlos Navas, & Edwin Rajotte.

-Changes in gene expression associated with thermal tolerance in R. padi: To explore potential molecular mechanisms underlying the increased heat tolerance of R. padi infected with BYDV-PAV, I) we extracted mRNA from virus-free R. padi and viruliferous aphids exposed to room temperature (23 °C) and heat stress (CTMax), three replicates per treatment factor. Twenty adults (4-day old) provide a good amount of sample for Illumina HiSeq. 2500. R. padi is a small aphid (1.5-2.5mm) and its necessary to ensure a good amount and high quality RNA for sequencing. Mitzy Porras conducted CTMax experiments and RNA extractions, Juan A Raygoza analyzed the transcriptomes.
II) To quantify the expression levels of candidate genes identified from the transcriptomic analysis we followed the same procedure as described above (I) to keep the consistency with the previous part of our experiment. We extracted RNA and synthetized cDNA to conduct qRT-PCR. Each treatment factor was replicated eight times and compared against two housekeeping genes. Our replication allowed to clearly observe how several heat shock proteins were highly upregulated in viruliferous aphids. Mitzy Porras conducted CTMax experiments, RNA extraction, cDNA synthesis, and qRT-PCR. Mitzy Porras & Andres Sandoval-Mojica constructed primers and analyzed data.

-Temperature and viral infection modulate interspecific competition between aphids: We recorded the lifespan and the fecundity per individual. Each factor-combination was replicated 15 times using new plants and the vectors Rhopalosiphum padi and R. maidis. Measurements were taken by Mitzy Porras, Edwin Rajotte, D. Smilnak, & M. M. Douglas.

-Supplementary Information-Effects of viral infection on the lethal thermal dose 50: An individual adult (4-day old; aphids were grown on 20 different plants per infection treatment) were placed on a metal pelt adapted to a ceramic hotplate inside automated thermal chamber and exposed to fifteen temperatures (18, 20, 22, 24, 26, 28, 30, 32, 34, 36, 38, 40, 42, 44, and 47 °C) for 56 min. We recorded data as survival yes or no. Measurements were taken by Mitzy Porras, Edwin Rajotte, & K. Kennedy.

-Supplementary Information-Effects of viral infection on the locomotor capacity: We measured the walking speed of virus-free and viruliferous aphids of each species. We recorded with a stopwatch the time it took to walk the length of a tube (10 cm length). Measurements were taken by Mitzy Porras, K. Kennedy, & W. Wenckus.

-Supplementary information- Behavioral thermal preference of aphids in an artificial arena: We measured the effect of BYDV strains on the thermal preference of both aphid species. We recorded aphid body temperature using a thermocouple thermometer with RS232 output data logger (VWR, Radnor, PA, USA). Measurements were taken by Mitzy Porras & W. Wenckus. The artificial thermal

arena was designed and constructed by Mitzy Porras; guidance: Sylvain Pincebourde & German Holguin.

Timing and spatial scale

-Virus effects on plant temperature-Thermal profile of host plants: Thermal profile of host plants: 13-15 June of 2015. Frequency: three times during the week. A wheat field in Rock Springs Experimental Station in central Pennsylvania (USA) (60m x 60m).

-Virus effects on plant temperature - Thermal characterization of virus-free and virus-infected plants under controlled conditions: 16-21 June of 2015. Thermal images were obtained between 9:00 and 11:00 am. Spatial scale: organismal, laboratory conditions, experiments conducted inside Conviron chambers (CMP 3244) at Penn State University.

-Interspecific competition and viral infection influence the spatial distribution of aphids: 17 June of 2014; frequency: one time measurements. Spatial scale: organismal, the experiments were conducted at the Pennsylvania State University Horticultural Facility (University Park, PA).

-Virus effects on thermal tolerance of aphids: August- September 2014. Frequency: every day. Spatial scale: organismal, laboratory conditions.

-Changes in gene expression associated with thermal tolerance in R. padi: November 2015-September 2016.RNA extractions were conducted in November 2015 and July-August of 2016. Transcriptomes were constructed from February to May of 2016. qRT-PCR was conducted in September of 2016.

-Temperature and viral infection modulate interspecific competition between aphids: May-2013 to June-2014. Frequency: mostly every day. Spatial scale: organismal, laboratory conditions.

-Supplementary Information-Effects of viral infection on the lethal thermal dose 50: 8- 28 June 2015. Frequency: measurements were taken three times per day for 20 days. Spatial scale: organismal, laboratory conditions.

-Supplementary Information-Effects of viral infection on the locomotor capacity: 5-12 December of 2015. Frequency: measurements were taken every 3h. Spatial scale: organismal, laboratory conditions.

-Supplementary information- Behavioral thermal preference of aphids in an artificial arena: 15 May to 13 June 2014. Frequency: measurements were taken every day in the morning. Spatial scale: organismal, laboratory conditions.

Data exclusions

-Virus effects on plant temperature - Thermal characterization of virus-free wheat plants under field conditions: No data were excluded from the analyses.

-Virus effects on plant temperature - Thermal characterization of virus-free and virus-infected plants under controlled conditions: No data were excluded from the analyses.

-Interspecific competition and viral infection influence the spatial distribution of aphids: No data were excluded from the analyses.

-Virus effects on thermal tolerance of aphids: No data were excluded from the analyses.

-Changes in gene expression associated with thermal tolerance in R. padi: No data were excluded from the analyses.

-Temperature and viral infection modulate interspecific competition between aphids: No data were excluded from the analyses.

-Supplementary Information-Effects of viral infection on the lethal thermal dose 50: No data were excluded from the analyses.

-Supplementary Information-Effects of viral infection on the locomotor capacity: No data were excluded from the analyses.

-Supplementary information- Behavioral thermal preference of aphids in an artificial arena: No data were excluded from the analyses.

Reproducibility

-Virus effects on plant temperature- Thermal characterization of virus-free wheat plants under field conditions: All attempts to repeat the experiment were successful.

-Virus effects on plant temperature - Thermal characterization of virus-free and virus-infected plants under controlled conditions: All attempts to repeat the experiment were successful.

-Interspecific competition and viral infection influence the spatial distribution of aphids: All attempts to repeat the experiment were successful.

-Virus effects on thermal tolerance of aphids: All attempts to repeat the experiment were successful.

-Changes in gene expression associated with thermal tolerance in R. padi: All attempts to repeat the experiment were successful after identifying the number of aphids need to extract a good amount of RNA for the transcriptomic analysis.

-Temperature and viral infection modulate interspecific competition between aphids: All attempts to repeat the experiment were successful.

-Supplementary Information-Effects of viral infection on the lethal thermal dose 50: All attempts to repeat the experiment were successful.

-Supplementary Information-Effects of viral infection on the locomotor capacity: All attempts to repeat the experiment were successful.

-Supplementary information- Behavioral thermal preference of aphids in an artificial arena: All attempts to repeat the experiment were successful.

Randomization

-Virus effects on plant temperature- Thermal characterization of virus-free wheat plants under field conditions: All plants used for the infrared pictures were randomly chosen in the wheat field.

-Virus effects on plant temperature - Thermal characterization of virus-free and virus-infected plants under controlled conditions: All plants were randomly chosen from the set of virus-free and virus-infected plants (180 plants for each treatment), their thermal pictures were randomly taken.

-Interspecific competition and viral infection influence the spatial distribution of aphids: plants and aphids used in the experiments were randomly chosen from small aphid colonies with 15-25 aphids.
-Virus effects on thermal tolerance of aphids: aphids used in the experiments were randomly chosen from small aphid colonies with 15-25 aphids.

-Changes in gene expression associated with thermal tolerance in R. padi: Changes in gene expression associated with thermal tolerance in R. padi aphids used in the experiments were randomly chosen. from small aphid colonies with 15-25 aphids.

-Temperature and viral infection modulate interspecific competition between aphids: All plants were randomly chosen from sets of 180 plants. Aphids used in the experiments were randomly chosen from small aphid colonies with 15-25 aphids.

-Supplementary Information-Effects of viral infection on the lethal thermal dose 50: aphids used in the experiments were randomly chosen from small aphid colonies with 15-25 aphids.

-Supplementary Information-Effects of viral infection on the locomotor capacity: aphids used in the experiments were randomly chosen from small aphid colonies with 15-25 aphids.

-Supplementary information- Behavioral thermal preference of aphids in an artificial arena: All plants and aphids used in the experiments were randomly chosen from small aphid colonies with 15-25 aphids.

Blinding

-Virus effects on plant temperature- Thermal characterization of virus-free wheat plants under field conditions: All plants used for the infrared pictures were randomly chosen in the wheat field (Spring wheat). Each time that we took a picture we calibrated the camera using a black body, a wet and a dry plant.

-Virus effects on plant temperature - Thermal characterization of virus-free and virus-infected plants under controlled conditions: All plants were randomly chosen from sets of virus-free and virus-infected plants; We did not assign any order to the treatments, then their thermal pictures were randomly taken. each picture had a wet and a dry plant as a control. Wet plants were sprayed with distilled water; dry plans were covered with Vaseline.

-Interspecific competition and viral infection influence the spatial distribution of aphids: All plants and aphids used in the experiments were at the same developmental stage and were randomly chosen. Plants were grown in individual cones (5cm diameter x 38cm height), under the same watering and environmental conditions. Aphid were collected in small colonies from small aphid colonies with 15-25 aphids.

-Virus effects on thermal tolerance of aphids: Aphids used in the experiments were at the same developmental stage (adult) and the same age (4-day old). Aphids randomly chosen from 20 small colonies cultured on wheat? plants in a growth chamber.

-Changes in gene expression associated with thermal tolerance in R. padi: the aphids used in the experiments were at the same developmental stage (adult) and the same age (4-day old). Aphids randomly chosen from 15 small colonies from small aphid colonies with 15-25 aphids.

-Temperature and viral infection modulate interspecific competition between aphids: Aphids used in the experiments were at the same developmental stage (adult) and the same age (4-day old). Aphids randomly chosen from 60 small colonies cultured on wheat? plants in a growth chamber. Plants were randomly chosen from small aphid colonies with 15-25 aphids.

-Supplementary Information-Effects of viral infection on the lethal thermal dose 50: Aphids used in the experiments were at the same developmental stage (adult) and the same age (4-day old). Aphids randomly chosen from 60 small colonies cultured on wheat? plants in a growth chamber.

-Supplementary Information-Effects of viral infection on the locomotor capacity: aphids used in the experiments were at the same developmental stage (adult) and the same age (4-day old). Aphids randomly chosen from 50 small colony-plants.

-Supplementary information- Behavioral thermal preference of aphids in an artificial arena: Aphids used in the experiments were at the same developmental stage (adult) and the same age (4-day old). Aphids randomly chosen from 50 from small aphid colonies with

15-25 aphids.

Did the study involve field work? ☒ Yes ☐ No

Field work, collection and transport

Field conditions	-Virus effects on plant temperature- Thermal characterization of virus-free wheat plants under field conditions: Wheat crop<br>-Interspecific competition and viral infection influence the spatial distribution of aphids: wheat plants in a raised bed with natural soil.
Location	-Virus effects on plant temperature- Thermal characterization of virus-free wheat plants under field conditions: Rock Springs Experimental Station, Penn State University, central Pennsylvania (USA).<br><br>-Interspecific competition and viral infection influence the spatial distribution of aphids: Horticulture Greenhouse Facility at Penn State University, Campus University Park. State College, PA.
Access and import/export	-Virus effects on plant temperature- Thermal characterization of virus-free wheat plants under field conditions: Rock Springs Experimental Station belongs to PennState University, we had free access to wheat crops at this Research Center.<br><br>-Interspecific competition and viral infection influence the spatial distribution of aphids: we had free access to the Horticulture Greenhouse Facility.
Disturbance	None

Reporting for specific materials, systems and methods

We require information from authors about some types of materials, experimental systems and methods used in many studies. Here, indicate whether each material, system or method listed is relevant to your study. If you are not sure if a list item applies to your research, read the appropriate section before selecting a response.

Materials & experimental systems

n/a	Involved in the study	
☒	☐	Antibodies
☒	☐	Eukaryotic cell lines
☒	☐	Palaeontology
☐	☒	Animals and other organisms
☒	☐	Human research participants
☒	☐	Clinical data

Methods

n/a	Involved in the study	
☒	☐	ChIP-seq
☒	☐	Flow cytometry
☒	☐	MRI-based neuroimaging

Animals and other organisms

Policy information about studies involving animals; ARRIVE guidelines recommended for reporting animal research

Laboratory animals	Rhopalsophum padi (Insecta: Hemiptera: Aphididae), R. maidis (Insecta: Hemiptera: Aphididae), Barley yellow dwarf virus strains BYDV-PAV and BYDV-RMV (Virus:Luteoviridae)
Wild animals	NA
Field-collected samples	The study did not involve wild animals.
Ethics oversight	aphis USDA approved our application work with aphids (R. padi and R. maidis) and barley yellow dwarf virus, permit number 526-141002-005.

Note that full information on the approval of the study protocol must also be provided in the manuscript.
